# Quantification of angiotensin II-regulated proteins in urine of patients with polycystic and other chronic kidney diseases by selected reaction monitoring

**DOI:** 10.1186/s12014-016-9117-x

**Published:** 2016-08-05

**Authors:** Ana Konvalinka, Ihor Batruch, Tomas Tokar, Apostolos Dimitromanolakis, Shelby Reid, Xuewen Song, York Pei, Andrei P. Drabovich, Eleftherios P. Diamandis, Igor Jurisica, James W. Scholey

**Affiliations:** 1 Division of Nephrology, Department of Medicine, Toronto General Hospital, University Health Network, University of Toronto, 11-PMB-189, 585 University Avenue, Toronto, ON M5G 2N2 Canada; 2 Toronto General Research Institute, University Health Network, Toronto, Canada; 3 Department of Laboratory Medicine and Pathobiology, Lunenfeld-Tanenbaum Research Institute, Mount Sinai Hospital, University of Toronto, Toronto, Canada; 4 Princess Margaret Cancer Centre, University Health Network, University of Toronto, Toronto, Canada; 5 Division of Genomic Medicine, University Health Network, University of Toronto, Toronto, Canada; 6 Department of Clinical Biochemistry, University Health Network, University of Toronto, Toronto, Canada; 7 Departments of Medical Biophysics and Computer Science, University Health Network, University of Toronto, Toronto, Canada

**Keywords:** Angiotensin II, Renin angiotensin system, Polycystic kidney disease, Autosomal dominant polycystic kidney disease, Selected reaction monitoring, Proteomics, Urine, Chronic kidney disease

## Abstract

**Background:**

Angiotensin-II (Ang II) mediates progression of autosomal-dominant polycystic kidney disease (ADPKD) and other chronic kidney diseases (CKD). However, markers of kidney Ang II activity are lacking. We previously defined 83 Ang II-regulated proteins in vitro, which reflected kidney Ang II activity in vivo.

**Methods:**

In this study, we developed selected reaction monitoring (SRM) assays for quantification of Ang II-regulated proteins in urine of ADPKD and CKD patients. We demonstrated that 47 of 83 Ang II-regulated transcripts were differentially expressed in cystic compared to normal kidney tissue. We then developed SRM assays for 18 Ang II-regulated proteins overexpressed in cysts and/or secreted in urine. Methods that yielded CV ≤ 6 % for control proteins, and recovery ~100 % were selected. Heavy-labeled peptides corresponding to 13 identified Ang II-regulated peptides were spiked into urine samples of 17 ADPKD patients, 9 patients with CKD predicted to have high kidney Ang II activity and 11 healthy subjects. Samples were then digested and analyzed on triple-quadrupole mass spectrometer in duplicates.

**Resluts:**

Calibration curves demonstrated linearity (R^2^ > 0.99) and within-run CVs < 9 % in the concentration range of 7/13 peptides. Peptide concentrations were normalized by urine creatinine. Deamidated peptide forms were monitored, and accounted for <15 % of the final concentrations. Urine excretion rates of proteins BST1, LAMB2, LYPA1, RHOB and TSP1 were significantly different (p < 0.05, one-way ANOVA) between patients with CKD, those with ADPKD and healthy controls. Urine protein excretion rates were highest in CKD patients and lowest in ADPKD patients. Univariate analysis demonstrated significant association between urine protein excretion rates of most proteins and disease group (p < 0.05, ANOVA) as well as sex (p < 0.05, unpaired t test). Multivariate analysis across protein concentration, age and sex demonstrated good separation between ADPKD and CKD patients.

**Conclusions:**

We have optimized methods for quantification of Ang II-regulated proteins, and we demonstrated that they reflected differences in underlying kidney disease in this pilot study. High urine excretion of Ang II-regulated proteins in CKD patients likely reflects high kidney Ang II activity. Low excretion in ADPKD appears related to lack of communication between cysts and tubules. Future studies will determine whether urine excretion rate of Ang II-regulated proteins correlates with kidney Ang II activity in larger cohorts of chronic kidney disease patients.

**Electronic supplementary material:**

The online version of this article (doi:10.1186/s12014-016-9117-x) contains supplementary material, which is available to authorized users.

## Background

Renin angiotensin system (RAS) activity within the kidney plays a major role in the progression of most chronic kidney diseases, including polycystic kidney disease. The main effector of the RAS is angiotensin II (Ang II), an octapeptide that exerts diverse adverse hemodynamic and non-hemodynamic effects on the kidney, including increased sodium reabsorption, increased vasoconstriction, increased inflammation, oxidative stress, and fibrosis [1–7]. In autosomal-dominant polycystic kidney disease (ADPKD), a main form of polycystic kidney disease, Ang II plays a role in the pathogenesis of hypertension, and contributes to cyst growth [8, 9]. Cyst growth measured by magnetic resonance imaging (MRI) has been associated with ADPKD progression [10, 11]. Blockade of the RAS is the mainstay treatment of chronic kidney disease (CKD), and it has been shown to slow down progression of ADPKD [12–15]. However, overzealous RAS blockade may have serious adverse effects [16, 17]. Thus, monitoring of Ang II activity within the kidney would enable adequate dose-adjustments of RAS blockers, avoid the consequences of overly aggressive RAS blockade, and help estimate patient’s risk of kidney disease progression. Unfortunately, specific markers of kidney Ang II activity are lacking.

In clinical practice, it would be ideal to monitor markers of RAS and Ang II activity in a non-invasive biofluid such as urine. Recent literature addressing markers of RAS activity in the kidney has focused primarily on measuring urine excretion rates of angiotensinogen. Angiotensinogen is a precursor protein that is enzymatically cleaved by renin to generate angiotensin I, which is subsequently cleaved by angiotensin converting enzyme (ACE) to generate Ang II. Several studies have reported increased urinary angiotensinogen excretion in patients with CKD, and in a few studies angiotensinogen excretion correlated with CKD progression [18–20]. Studies of patients with ADPKD also echoed the potential link between urine angiotensinogen excretion and factors associated with progression, such as hypertension, eGFR and total kidney volume [21, 22]. Plasma angiotensinogen levels are poised near the Km for cleavage by renin, so that alteration of angiotensinogen levels also affects the formation of Ang II [23]. Nonetheless, kidney RAS is complex, and simple substrate measurements may not be indicative of true RAS bioactivity. This was most clearly demonstrated by Matsusaka and colleagues, who showed that kidney angiotensinogen levels can indeed be measured in urine of mice, however, it is the liver angiotensinogen that is enzymatically cleaved in proximal tubules of the kidney to generate Ang II [24, 25]. There is thus rationale to search for more specific markers of Ang II activity in the kidney, and we aim to fulfill this unmet clinical need.

We have previously identified 83 proteins differentially regulated by Ang II in primary human proximal tubular cells [26]. We verified differential expression of 18 of these 83 proteins by using selected reaction monitoring (SRM) in primary tubular cell lysates. Subsequently, we demonstrated that these Ang II-regulated proteins were differentially expressed in kidney tissue of two distinct mouse models of chronic kidney disease, and at least one top candidate protein was measurable in urine, and its excretion rate correlated with kidney Ang II levels [26, 27]. It is thus tempting to postulate that kidney tissue expression of these Ang II-regulated proteins may reflect disease severity in human forms of chronic kidney disease. Furthermore, a subgroup of these Ang II-regulated proteins may be excreted in urine, and their urinary excretion rate may correlate with disease severity and Ang II activity in the kidney. The aim of this study was to develop SRM-based assays for quantification of Ang II-regulated proteins in human urine. For method development and initial pilot study, we utilized urine samples of healthy subjects and patients with ADPKD and other CKDs.

## Methods

### Reagents

The reagents used in this study were as follows. Acetonitrile (HPLC grade) and ammonium bicarbonate were from Fisher Scientific (NJ, USA). Amicon^®^ Ultra 3KDa centrifugal filters and formic acid were from EMD Millipore (Merck KGaA, Darmstadt, Germany). Proteomics-grade trypsin, dithiotreitol, iodoacetamide, and bovine serum albumin (>98 % pure) were from Sigma-Aldrich (Oakville, ON, Canada). Proteomics-grade trypsin/lys-C mix was from Promega (Madison, WI, USA). Ultrapure grade urea was from Amresco (Solon, OH, USA). Ultrapure water was from Milli-Q (Millipore, Molsheim, France). Crude unlabeled, crude isotopically labeled (lysine: 6 × 13C, 2 × 15N, arginine: 8 × 13C, 2 × 15N) and purified isotopically labeled peptides were from JPT Peptide Technologies (Berlin, Germany). OMIX C18 10 μl tips were from Agilent Technologies (Lake Forest, CA, USA).

### Samples

Second morning spot urine samples (10–50 mL) were collected from 11 healthy volunteers, 17 patients with autosomal dominant polycystic kidney disease (ADPKD), and 9 patients with non-ADPKD chronic kidney disease (CKD). These samples were centrifuged at 1000*g* for 10 min at room temperature. The supernatants were transferred to a fresh tube, and immediately frozen at −80 °C. After thawing in 37 °C bath, urine samples were centrifuged at 2000*g* for 10 min at room temperature and debris was discarded. The supernatants were transferred to a fresh tube and the urinary protein concentration was measured by a clinical benzethonium chloride-based assay (Roche). Urine creatinine concentrations were determined by Jaffé colorimetric assay.

### Study population

Patients with ADPKD and CKD were followed in the kidney disease clinic by Dr. York Pei at Toronto General Hospital in Toronto. Patients were selected from a research database, based on availability of urine sample. Healthy volunteers were selected as controls. Patients’ and controls’ characteristics are presented in Additional file 1: Table S1.

### Selection of proteins and peptides for SRM method development

SRM methods were developed for those Ang II-regulated proteins with the highest likelihood of being found in urine and those previously monitored by SRM [26]. To determine presence in urine, we first searched publically available urine databases including: (1) Max-Planck Unified Proteome Database: http://www.mapuproteome.com/urine/; (2) a publication documenting >400 proteins in human urine [28]; (3) Peptide Atlas Database (http://www.peptideatlas.org); and (4) ExoCarta (http://www.exocarta.org). We also searched PubMed for publications explicitly documenting the proteins of interest in urine. Ten Ang II-regulated proteins were discovered in urine when applying the search algorithm described (TSP1, GLUL, SPARC, TGFBR2, BST1, LYPLA1, LAMB2, HO1, RHOB and DBNL). Finally, we selected six additional Ang II-regulated proteins (PDCD4, ARHGEF2, VCPIP1, DNAJB4, TXNIP, and PHLDA1) that we previously monitored in human proximal tubular cells [26], and two additional Ang II-regulated proteins predicted to be secretory based on the signal sequence or transmembrane region (Signal IP algorithm; Human Protein Atlas: http://www.proteinatlas.org) (EGFR and RBM3). Most of the selected proteins were differentially expressed at mRNA level in cysts of patients with ADPKD, compared to minimally cystic or normal kidney tissue (as will be discussed in the results). Thus 18 proteins were selected for SRM method development (Table 1).

**Table 1:** Proteins selected for development of SRM assays in urine

Protein/uniprot ID	Uniprot ID	Peptide amino acid sequence
Heme oxygenase-1 (HO-1)^ab^	P09601	VQDSAPVETPR TEPELLVAHAYTR
Thrombospondin-1 (TSP-1)^ab^	P07996	**TIVTTLQDSIR** **GGVNDFQGVLQNVR**
Glutamate-ammonia ligase(GLUL)^a^	P15104	LTGFHETSNINDFSAGVANR TCLLNETGDEPFQYK **LVLCEVFK**
Ras homologue family member B (RHOB)^ab^	P62745	LVVVGDGACGK **IQAYDYLECSAK**
Osteonectin (SPARC)^a^	P09486	YIPPCLDSELTEFPLR FFETCDLDNDK
Transforming growth factor beta, receptor II (TGFBR2)^a^	P37173	LDPTLSVDDLANSGQVGTAR **IFPYEEYASWK**
Bone marrow stromal cell antigen 1 (BST1)^a^	Q10588	**GFFADYEIPNLQK** AGLIIPLFLVLASR
Lysophospholipase I (LYPA1)^a^	O75608|	ASFPQGPIGGANR **LAGVTALSCWLPLR**
Laminin, beta 2 (LAMB2)^a^	P55268	LQEGQTLEFLVASVPK **GSCYPATGDLLVGR**
Drebrin-like (DBNL)^ab^	Q9UJU6	**VAGTGEGGLEEMVEELNSGK** FQDVGPQAPVGSVYQK
Programmed cell death 4 (PDCD4)^b^	Q53EL6	APQLVGQFIAR SGVPVLAVSLALEGK
Thioredoxin interacting protein (TXNIP)^bc^	Q9H3M7	SFEVVFNDPEK HTYLANGQTK
Pleckstrin-homology like domain, Family A, Member 1(PHLDA1)^bc^	Q8WV24	AAGNGEAEPSGGPSYAGR YMYFTVVMAFGK
Rho/Rac guanine nucleotide exchange factor 2 (ARHGEF2)^b^	Q92974	DLLVGPGVELLLTPR ELLSNVDEGIYQLEK
Hsp40 homologue, Subfamily B, Member 4 (DNAJB4)^bc^	Q9UDY4	IIGYGLPFPK **EALCGCSINVPTLDGR**
Valosin containing protein (p97)/p47 complex interacting protein (VCPIP1)^b^	Q96JH7	TEPSVFTASSSNSELIR **SSGDYSATFLPGLIPAEK**
Epidermal growth factor receptor (EGFR)^c^	P00533	**EISDGDVIISGNK** ELVEPLTPSGEAPNQALLR
RNA binding motif, protein 3 (RBM3)^c^	P98179	YYDSRPGGYGYGYGR **GGGDQGYGSGR**

Bold peptides were detected and selected for absolute quantification in human urine

^a^Proteins definitively found in urine in prior studies

^b^Proteins previously monitored by SRM in our study

^c^Proteins predicted to be secretory based on computational algorithm (SignalIP)

**Fig. 1:**
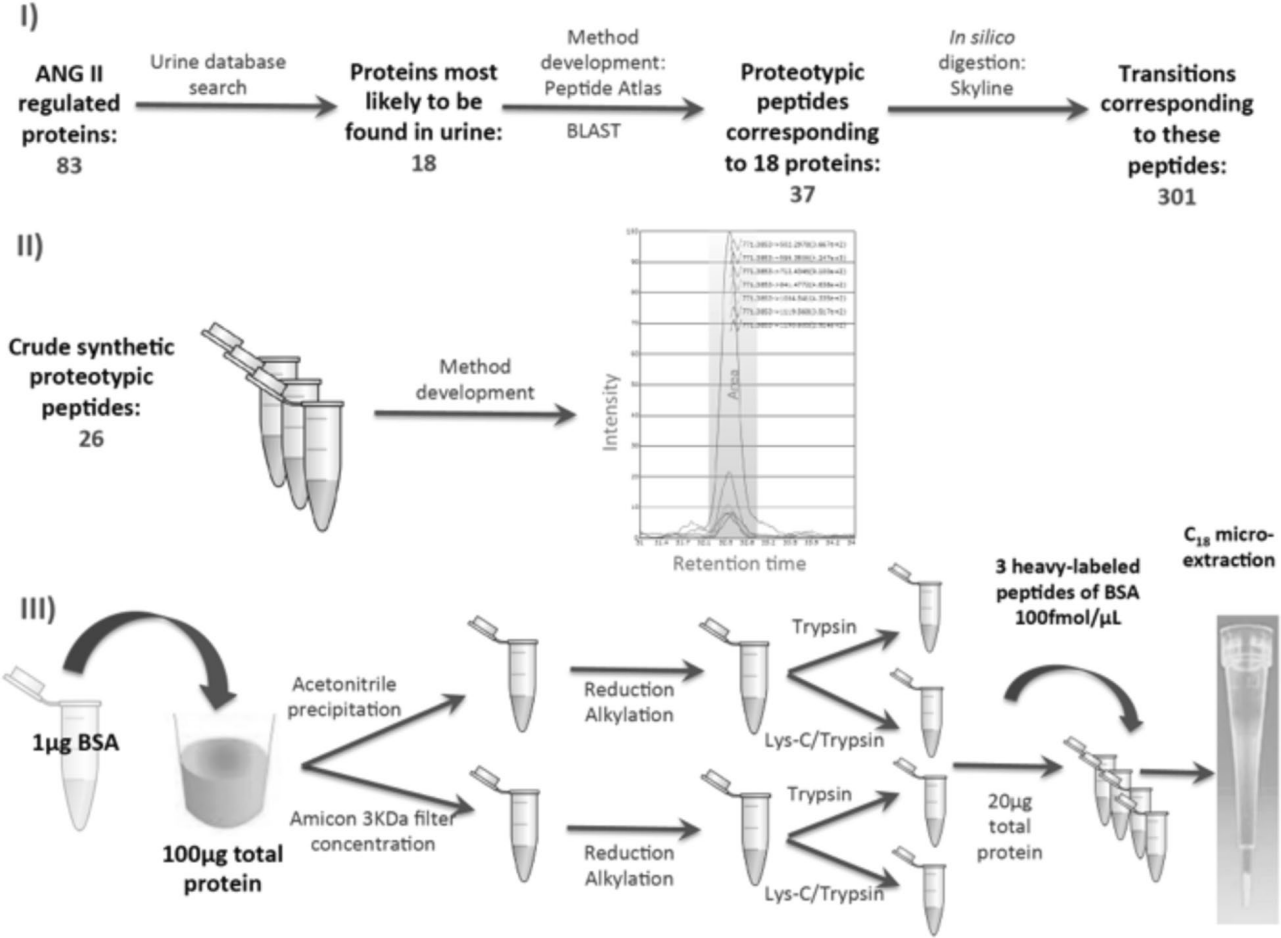
Steps in SRM method development. **I** ANG II-regulated proteins most likely to be found in urine were selected for SRM method development. Most highly observable proteotypic (unique) peptides were selected from Peptide Atlas, and searched with the protein BLAST (http://blast.ncbi.nlm.nih.gov/Blast.cgi) to ensure their uniqueness. Transitions were then selected by in silico digestion in Skyline. **II** To develop SRM methods we purchased 26 crude, synthetic, unlabeled peptides, which were used to determine retention time and order of transitions. 11 additional peptides had methods previously developed in human primary cells. **III** To determine reproducibility and recovery of sample preparation, we spiked 1 µg of BSA protein into urine containing 100 µg of total protein. We examined four different sample preparation protocols: ACN precipitation, Amicon filter concentration, digestion with trypsin alone or digestion with Lys-C and trypsin. Finally we spiked in three heavy isotope-labeled peptides corresponding to BSA, and fractions containing 20 µg total protein were subjected to C_18_ microextraction, prior to injection on triple quadrupole

The experimental design is presented in Fig. 1. Two to three proteotypic peptides were selected for each of the 18 proteins. Details regarding peptide selection, method development and data analysis have been published before [29]. The most highly observable peptides based on the +2 ions from Peptide Atlas were selected. Fully tryptic and doubly charged peptides with 7–20 amino acids were chosen. Peptides with methionine, tryptophan, and N-terminal cysteine residues were avoided, whenever possible. All peptides were also analyzed with the protein Basic Local Alignment Search Tool (BLAST) (http://blast.ncbi.nlm.nih.gov/Blast.cgi) to ensure that peptides were proteotypic to each protein. In this way we compiled a list of 37 proteotypic peptides for SRM method development. Skyline software was then used to select transitions containing y ions from y + 3 to last ion-1 for each peptide in silico. We selected 301 transitions corresponding to our proteotypic peptides (5–8 transitions per peptide).

### SRM method development

In the second step of method development (Fig. 1), we determined the retention time and the order of transitions by intensity for each of the 37 peptides. In case of 11/37 peptides, we had established retention times and order of transition intensities from the previous SRM experiments [26]. For the remaining 26/37 peptides, we purchased corresponding crude unlabeled synthetic peptides that were subjected to reduction, alkylation, and trypsin digestion, as recommended by the manufacturer. The peptide mixture was subsequently concentrated by the OMIX C18 tip and analyzed by LC–MS/MS on triple quadrupole instrument, as described below.

### SRM method optimization in human urine

In the third step of method development, we established and optimized a protocol for reproducible recovery of Ang II-regulated peptides. We started from healthy control urine aliquots (four per urine sample), each containing 100 μg of total protein. To assess reproducibility and recovery, we spiked in non-human protein, bovine serum albumin (BSA) at a known concentration of 1 μg (1 % of total protein). We then compared protein precipitation with acetonitrile to protein concentration with Amicon^®^ Ultra 3KDa centrifugal filters (Fig. 1). Urine samples containing 100 μg of total protein and precipitated overnight with acetonitrile at a ratio of 1:9 (v/v), were then centrifuged at 3220*g* for 30 min at 4 °C. The pellets were washed with acetonitrile twice, then air-dried. Pellets were then resuspended in 100 μL of denaturing buffer consisting of 8 M urea and 0.1 M ammonium bicarbonate. Denatured samples were reduced with 20 mM dithiotreitol in 50 mM ammonium bicarbonate at 37 °C for 30 min. Reduced samples were alkylated with 80 mM iodoacetamide in 50 mM ammonium bicarbonate at room temperature for 30 min in the dark.

In parallel to precipitation with acetonitrile, we concentrated urine proteins with Amicon^®^ Ultra 3KDa centrifugal filters. We started from urine samples containing 100 μg of total protein that were centrifuged in individual filters at 3220*g* for 60 min. Buffer exchange with 8 M urea was performed and filters were centrifuged for additional 60 min. Finally, 100–200 μL of retentate was collected and subjected to reduction and alkylation as above.

Following reduction and alkylation, we compared protein digestion with trypsin to Lys-C/trypsin mix. Half of the aliquots precipitated with acetonitrile or concentrated with Amicon^®^ filters were subjected to trypsin digestion (1:50 w/w) at 37 °C overnight. The second half of the aliquots were treated with Lys-C/trypsin (1:50 w/w) and incubated at 37 °C for 3 h. Urea concentration in aliquots was then reduced to 2 M by the addition of 0.1 M ammonium bicarbonate, in order to enable trypsin digestion. Samples were then incubated at 37 °C overnight. In all cases, trypsin digestion was stopped by the addition of 1 % (v/v) formic acid. Samples were then vortexed and separated into aliquots containing 20 μg of total protein. They were subsequently frozen at −20 °C until further analysis.

After thawing of individual aliquots containing 20 μg of total protein, we spiked in three crude heavy isotope labeled proteotypic peptides of BSA at a concentration of 100 fmol/μL. Samples were then desalted and concentrated using OMIX C18 10 μl tips.

### SRM-based detection of Ang II-regulated proteins in human urine

Having optimized the methods for urine SRM based on the recovery and reproducibility of BSA peptides, we used these methods to monitor 37 Ang II-regulated peptides in two healthy urine samples, and nine urine samples from patients with ADPKD. The retention time and order of transition intensities determined during the analysis of crude unlabeled peptides was used for SRM scheduling, and to select the correct peptide peaks.

**Fig. 2:**
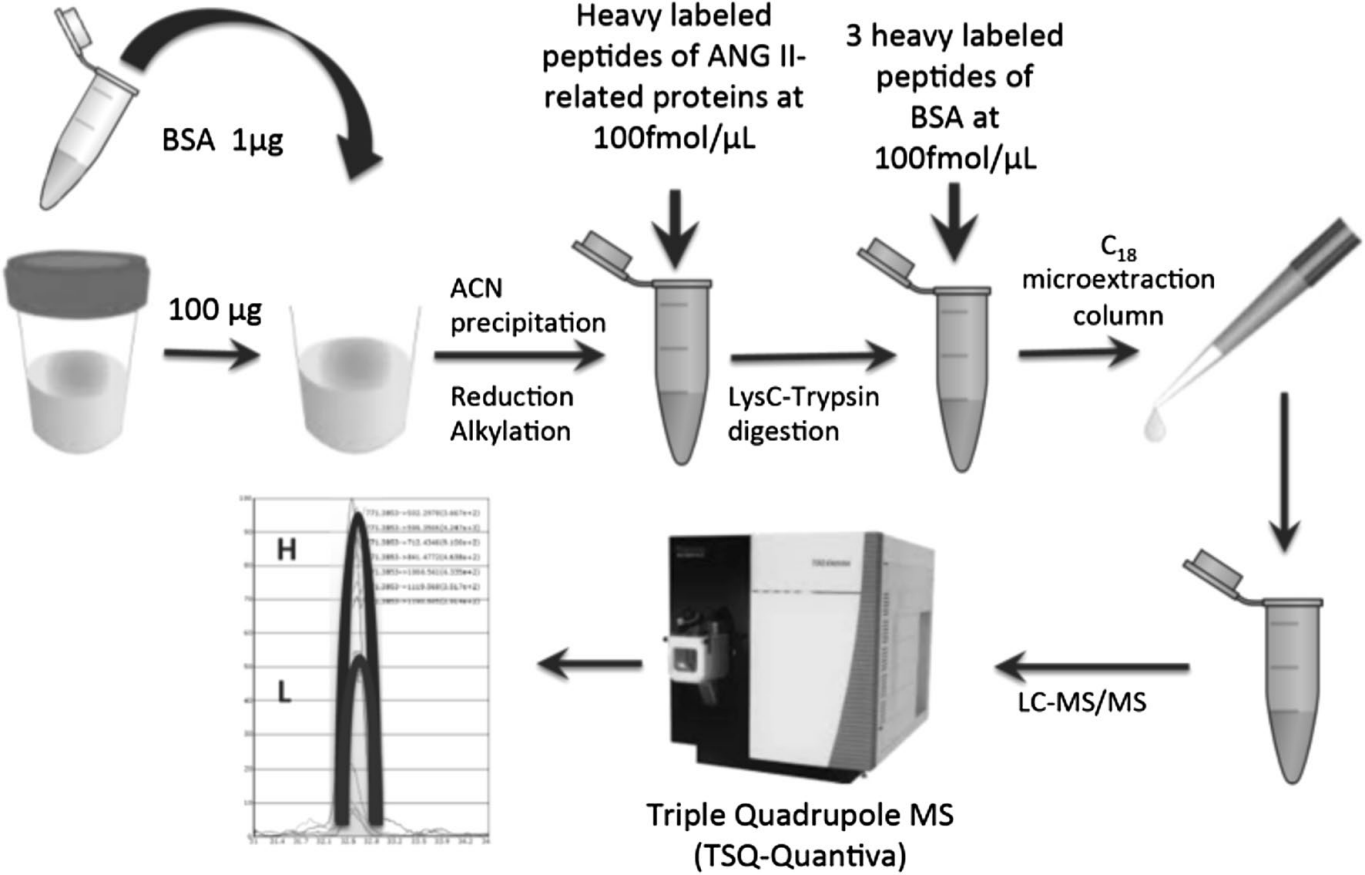
Absolute quantification of Ang II-regulated proteins in human urine samples. *ACN* acetonitrile, *BSA* bovine serum albumin, *Heavy labeled* heavy isotope-labeled peptide

We successfully identified 13 of 37 peptides in all urine samples (Table 1), and we then purchased the corresponding purified 13 heavy-labeled peptides with a trypsin-cleavable tag, to correct for digestion efficiency. Samples were then prepared as shown in Fig. 2. All samples were analyzed on the same day in duplicates, to assess within-run precision, which was expressed as mean CV of all samples, for each individual peptide. In addition, we assessed between-day precision in all urine samples, which included thawing of a new sample aliquot of the same urine sample on a different day, spiking in of BSA, precipitation of proteins, digestion, C_18_ microextraction, and analysis by LC-MS. This addressed variation due to sample preparation. Between-day precision was expressed as the mean CV of all samples analyzed on different days for each individual peptide. The samples were processed and run on mass spectrometry instrument in random order. Finally, calibration curves were generated, by spiking heavy-labeled peptides into the same urine sample at concentrations: 1000, 100, 10, 1, 0.1, and 0 fmol/μL. Blank sample contained equal volume of buffer used to suspend heavy-labeled peptides. Limit of detection (LOD) and limit of quantification (LOQ) were calculated for each peptide. LOD was calculated by taking the average of blank injections and adding three standard deviations to the mean. LOQ was calculated by taking the average of blank injections and adding 10 standard deviations to the mean.

### Liquid chromatography mass spectrometry parameters

Concentrated peptides were loaded in a volume of 18 μL onto a 3.3 cm pre-column (C18, 5 μm) and eluted on a 15 cm analytical column (C18, 3 μm). We used the following parameters: Flow rate = 300 nl/min during gradient, C18 material: Agilent Pursuit 5 μm for precolumn, 3 μm for analytical column; Precolumn ID: 150 μm × 3.2 mm; Analytical column ID: 75 μm × 15 cm. The reversed-phase liquid chromatography (EASY-nLC, Proxeon, Odense, Denmark) was coupled to a triple-quadrupole mass spectrometer (TSQ Quantiva, Thermo Fisher Scientific Inc., San Jose, CA) using a nanoelectrospray ionization source, as previously described [29–31]. Peptides were separated over 60 min, with a previously optimized 3-step gradient. We used Buffer A containing 0.1 % formic acid, and Buffer B containing acetonitrile and 0.1 % formic acid. In the first step of method development, 5–6 peptides and about 50 transitions were included in each survey SRM method and run in a non-scheduled mode with 20 ms scan time per transition, on a 60-min gradient. Q1 was set to 0.2 Thompson FWHM (Th, Full Width Half Maximum), Q3 to 0.7 Th FWHM, and Q2 pressure was set to 1.5 mTorr. The instrument was run in a positive ion mode, with collision energies predicted by Skyline software, according to the following formula: CE = 0.03 × (precursor *m/z*) + 2.905 [32]. Reproducibility of SRM signal was ensured by running a QC solution of 1 fmol/μL BSA every five runs.

After determining the retention times of our peptides of interest, we scheduled 10–15 peptides and approximately 80 transitions in each SRM method, with 20 ms scan time per transition, on a 60-min gradient. The other instrument parameters were unchanged. Final optimizations included increasing Q1 to 0.4 Th, decreasing scan time to 10 ms for abundant peptides, and increasing it to 30 ms for low abundance peptides.

Raw files recorded for each sample were analyzed using Skyline software [33], and CSV files with peptide areas were extracted. All peptides were manually inspected.

### Study of chemical modifications

In addition to monitoring unmodified forms of Ang II-regulated peptides, we examined for presence of oxidized methionines and deamidated asparagines and glutamines. Urine samples from healthy subjects and ADPKD patients were first examined for the presence of these forms, and we subsequently monitored modified heavy and light (naturally occurring) forms that demonstrated significant abundance. Peptides with modifications and transitions monitored are displayed in Additional file 1: Table S2.

### Data analysis

Skyline was used to verify the integration of chromatograms, and to calculate peptide areas. Light/heavy peak area intensity ratios were used to calculate concentrations of peptides monitored, by using the equation: light/heavy peptide ratio × heavy peptide concentration (fmol/μL) × volume of heavy peptides (μL)/(urine creatinine (μmol) × dilution factor). Recovery of BSA peptides was calculated as: observed BSA amount/spiked-in BSA amount × 100 %. Observed BSA amount was calculated from: light/heavy peptide ratio × heavy peptide concentration (fmol/μL) × volume of heavy peptides (μL).

Protein concentrations were first normalized by log2 transformation and subsequent subtraction of the log2 transformed concentrations of creatinine. For each of the proteins two sample t test was conducted to assess the significance of difference in the protein concentration between sexes (R’s function t.test). For each protein Pearson’s coefficient of correlation was calculated to evaluate correlation between concentration of the protein and age (R’s function cor). Significance of the correlation was then evaluated by cumulative distribution function of t distribution with n-2 degrees of freedom, where n is the number of measurements (R’s function cor.test). To assess the significance of differences of the protein concentration among sample types, for each protein we conducted ANOVA (R’s function aov), followed by Tukey’s range test (a. k. a. Tukey’s honest significance differences) to assess significance of differences between individual pairs of sample types (R’s function TukeyHSD). Finally multiple correspondence analysis (MCA) was performed on protein concentration and clinical data including sex and age, where all protein concentrations as well as age were first discretized into four levels each, in a way that preserves equal frequencies between levels. All steps were performed in R version 3.1.2. Two-tailed p value <0.05 was considered significant. GraphPad Prism software version 5 (GraphPad Software, Inc. La Jolla, CA) was used to display calibration curves and calculate regression equations.

For analyses of mRNA expression of Ang II-regulated proteins in polycystic kidneys compared to normal or minimally cystic kidney tissue, we used publicly available data from a previous study, and we selected all those Ang II-regulated transcripts with statistically significant expression determined by significance analysis of microarrays (SAM) with FDR < 0.05 between cystic and normal kidney tissues, and we displayed them in a heatmap. The expression of Ang II-regulated genes was compared to the whole microarray gene set by χ^2^ test.

Protein–protein interaction (PPI) partners of the Ang II-regulated proteins were first identified using Integrated Interactions Database (26516188), IID v2016-03 (http://ophid.utoronto.ca/iid/), selecting only experimentally validated partners (including orthologous interaction evidence). Ang II-regulated proteins and their PPI partners were then subjected to pathway enrichment analysis. Resulting network was visualized using NAViGaTOR v 2.3 (http://ophid.utoronto.ca/navigator) (1937718), and the corresponding XML file can be found at: http://www.cs.utoronto.ca/~juris/data/ClinProteom16/.

Considering the proteins in the network, we used pathDIP v1.0 (http://ophid.utoronto.ca/pathDIP) to perform pathway enrichment analysis across pathway variants gathered from major public pathways databases. List of 60 most enriched pathways were visualized by the barplot.

## Results

### Expression of Ang II-regulated genes in kidneys of patients with ADPKD

**Fig. 3:**
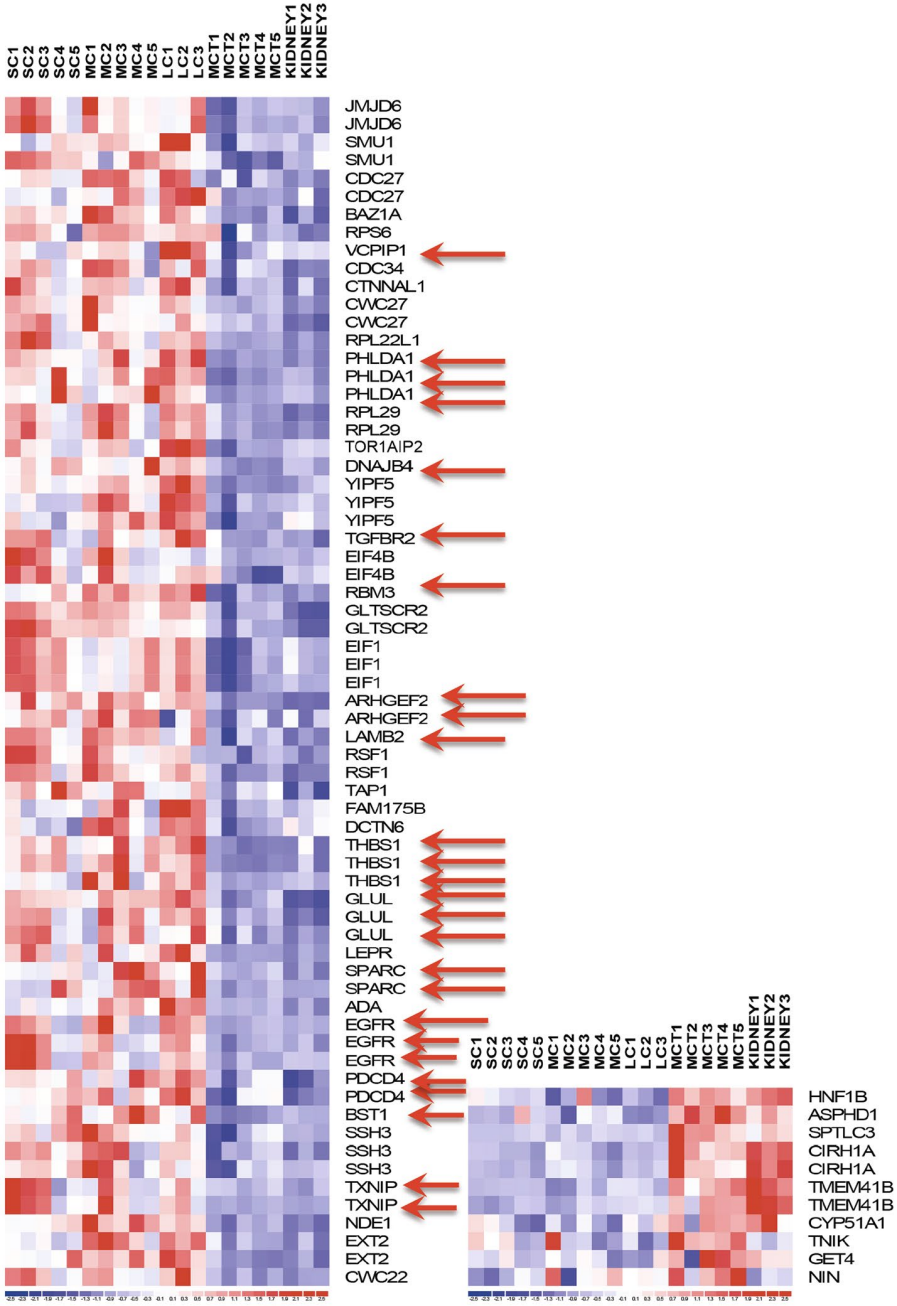
Heatmap of significant differentially expressed Ang II-regulated genes in cystic versus normal kidney tissues. *Each column* represents one tissue sample, and *each row* represents one gene. Because of several probes, some genes may occupy more than one row. Of 83 genes evaluated, 47 were differentially expressed (SAM, FDR < 0.05), as shown here. *Red colour* indicates upregulation and blue indicates downregulation. *Red arrows* point to 14 of 18 genes selected for SRM methods. *SC* small cysts, *MC* medium cysts, *LC* large cysts, *MCT* minimally cystic tissue, *Kidney* normal kidney tissue

We had previously identified 83 proteins differentially regulated by Ang II in primary human proximal tubular cells [26]. We first examined the expression of these proteins in kidneys of patients with ADPKD, a type of kidney disease in which progression has been linked to Ang II activity. A previous study examined gene expression in cysts of patients with ADPKD, compared to minimally cystic or normal kidney tissue [34]. We found that 47 out of 83 (57 %) Ang II regulated proteins significantly (SAM, FDR < 0.05) segregated cystic from normal tissues at the level of gene expression (Fig. 3). The number of Ang II-regulated genes found to be differentially expressed in cystic tissue was much higher than would be predicted by chance alone (χ^2^ = 108.2, p = 0, for the difference in proportion of differentially expressed genes in Ang II gene set compared to the whole Affymetrix set that contained 47,400 genes). We then developed SRM methods for quantification of 18 Ang II regulated proteins, and 14 of these were significantly upregulated in cystic kidney tissue compared to normal tissue (Fig. 3).

### Development of an optimized method for urine SRM analysis

The steps involved in SRM method development are described in methods and displayed in Fig. 1. We first selected 18 Ang II-regulated proteins most likely to be secreted in urine, found to be upregulated in cystic kidney tissue, and those we had previously monitored by SRM in human proximal tubular cell lysates (as described in methods). The complete list of proteins and peptides is shown in Table 1, and modifications and *m/z* values of peptides monitored are listed in Additional file 1: Table S2. We next used 26 crude unlabeled synthetic peptides (JPT Peptide Technologies) to determine the retention times and order of transition intensities, and these are displayed in Additional file 1: Table S3, together with the previously determined retention times for the remaining 11 peptides.

In the next step, we spiked in BSA protein prior to urine protein concentration, to determine method reproducibility and recovery. We processed two urine samples (1 female and 1 male; subjects 1 and 2 in Additional file 1: Table S1) with four different methods and compared peptide concentrations. These four methods included combinations of (1) protein precipitation using 3 kDa-filter (Amicon) or acetonitrile, and (2) protein digestion with Lys-C/trypsin or trypsin alone (Fig. 1). After digestion, samples were divided into aliquots each containing 20 μg of total protein, and three heavy-labeled peptides (three proteotypic peptides of BSA) were spiked in at a concentration of 100 fmol/μl. Each aliquot was subjected to C18 microextraction and analyzed on triple-quadrupole mass spectrometer (TSQ-Quantiva). Three BSA peptides were used to assess reproducibility and recovery (Table 2). Technical triplicate CVs (same sample injected three times on the instrument) were ≤6 % for BSA peptides, for each method (Table 2). BSA protein recovery was calculated by averaging the recovery of three BSA peptides. The recovery was 119 % in sample 1 and 112 % in sample 2, when precipitated with acetonitrile and digested with LysC/trypsin. Amicon filter concentration followed by LysC/trypsin digestion yielded BSA recovery of 171 % for sample 1 and 140 % for sample 2. Recovery >100 % may be explained by inaccurate quantification of spiked-in BSA, or perhaps by the carryover effect of BSA peptides used as QC. Trypsin digestion demonstrated inferior results. Acetonitrile precipitation followed by trypsin digestion demonstrated BSA recovery of 67 % for urine 1 and 64 % for urine 2. Amicon filter concentration followed by LysC/trypsin digestion yielded BSA recovery of 63 % for sample 1 and 44 % for sample 2. Protein precipitation with acetonitrile followed by Lys-C/trypsin digestion enabled detection of most Ang II-regulated peptides (13 of 37) in urine samples 1 and 2. Given the detection of the highest number of peptides of interest, we then applied acetonitrile precipitation combined with Lys-C/trypsin digestion to analyze urine samples from patients with ADPKD.

**Table 2:** Recovery and coefficients of variation (CVs) of 3 pmol of BSA protein and its proteotypic peptides in technical triplicates of two urine samples, utilizing four methods as described in the text

Protein/peptides	Mean recovery—urine 1 (pmol)^a^	CV (%)	Mean recovery—urine 2 (pmol)^a^	CV (%)
*Method 1*—*Amicon/LysC*-*Tryp*				
BSA				
LVNELTEFAK	6.137 ± 0.124	2.0	5.011 ± 0.08	1.7
HLVDEPQNLIK	6.242 ± 0.058	0.9	4.852 ± 0.059	1.2
LGEYGFQNALIVR	3.046 ± 0.056	1.8	2.770 ± 0.030	1.1
*Method 2*—*Amicon/Tryp*				
BSA				
LVNELTEFAK	2.061 ± 0.017	0.8	1.36 ± 0.064	4.7
HLVDEPQNLIK	2.317 ± 0.071	3.1	1.52 ± 0.037	2.4
LGEYGFQNALIVR	1.308 ± 0.011	0.8	1.044 ± 0.012	1.1
*Method 3*—*Acetonitrile/LysC*-*Tryp*				
BSA				
LVNELTEFAK	4.400 ± 0.029	0.7	3.980 ± 0.213	5.3
HLVDEPQNLIK	4.568 ± 0.119	2.2	3.984 ± 0.058	1.5
LGEYGFQNALIVR	1.768 ± 0.026	1.5	2.124 ± 0.013	0.6
*Method 4—Acetonitrile/Tryp*				
BSA				
LVNELTEFAK	2.00 ± 0.005	0.2	1.950 ± 0.010	0.5
HLVDEPQNLIK	3.46 ± 0.098	2.9	2.280 ± 0.130	5.8
LGEYGFQNALIVR	0.592 ± 0.005	0.9	1.535 ± 0.011	0.7

^a^Mean ± SD

### Ang II-regulated peptides detected in urine samples from controls and ADPKD patients

Using methods developed with the help of crude unlabeled peptides spiked into urine, we next monitored 37 peptides corresponding to 18 Ang II-regulated proteins in nine urine samples from ADPKD patients and two controls. We monitored both unmodified and modified forms (see below) of peptides. Using retention times and order of transitions derived with the use of crude peptides, we identified 13 peptides in all urine samples (Table 1). Peptide was considered present in urine if there was coelution of at least 4/6 or 5/7 transitions in the correct order and at the correct retention time. Heavy-labeled peptides for absolute quantification containing a trypsin-cleavable tag were thus purchased, corresponding to these 13 peptides. They were spiked in urine samples prior to tryptic digestion, and both heavy-labeled and light forms were monitored.

### Chemically modified peptides in urine samples from controls and ADPKD patients

In addition to unmodified peptides, we monitored peptide modifications including glutamine and asparagine deamidation, and methionine oxidation. Deamidation is a post-translational modification that may occur as a result of protein/peptide aging. Here, however, deamidation is most probably an artifact of sample preparation such as protein denaturation and treatment with acidic solutions (0.1 % formic acid). We first monitored deamidations in the control urine samples in order to determine whether these modifications contribute meaningfully to the total peptide area. Deamidation was present in both light and heavy-labeled peptides, whenever light peptides were detectable. It was detected in both light and heavy forms in the following peptides: GGV**N**D**N**F**Q**GVL**QN**VR (TSP-1), TIVTTL**Q**DSIR (TSP-1), GFFADYEIP**N**L**Q**K (BST1), and I**Q**AYDYLECSAK (RHOB). As a general rule, multiple deamidations in the same peptide did not significantly contribute to the overall peptide area (Additional file 1: Table S4). As noted in Additional file 1: Table S4, doubly and triply deamidated forms of peptide GGVNDNFQGVLQNVR accounted for <3 % of the total peptide area. Deamidations closer to the N-terminus of the peptide contributed to 50 % of the total peptide area, and only these modifications were selected for monitoring in all urine samples. The total H/L ratio was changed by 3–4 % when including all modified forms. In case of peptide GFFADYEIPNLQK, singly deamidated forms accounted for approximately 30–37 % of the total peptide areas, while doubly deamidated forms constituted 3.5–6 % of the unmodified peptide areas (Additional file 1: Table S4). The total H/L ratio was changed by 12 % due to the inclusion of deamidated forms. We decided to monitor all deamidated forms of this peptide. Deamidated peptide TIVTTLQDSIR accounted for 28 % of the total heavy-labeled peptide area, and 33–36 % of the total light peptide area. H/L ratio was changed by 7 % when including deamidated forms. Finally, deamidated form of peptide IQAYDYLECSAK was responsible for 20 % of the total heavy peptide area, and 10–30 % of the total light peptide area. H/L ratio was changed by 12 % when including deamidated forms (Table 3). Heavy peptides were stable, displaying predictable degree of deamidation across samples. In contrast, light peptides displayed higher and more variable degree of deamidation (Table 3). As a rule, deamidation contributed minimally to total peptide concentrations. The median % change in total peptide concentration as a result of deamidation was ≤7 % for TSP1 peptides and 12 % for BST1 and RHOB peptides.

**Table 3:** Deamidated peptides monitored and their contribution to the overall peptide areas and ratios

Protein/peptide	L/H ratio formula (non-deamidated formula on top, total on bottom)	Heavy peptide deamidation % (mean ± SD)	Light peptide deamidation % (mean ± SD)	Mean peptide % Conc. ∆ due to deamidation
TSP1/GGVNDNFQGVLQNVR	$\frac{808.9}{813.9}$	33 ± 1.2	39 ± 6.5	6.5
	$$\frac{808.9 + 809.4 + 809.4 + 809.4}{813.9 + 814.4 + 814.4 + 814.4}$$			
BST1/GFFADYEIPNLQK	$\frac{771.3}{775.3}$	35 ± 0.8	44 ± 3.8	12
	$$\frac{771.3 + 771.8 + 771.8 + 772.3}{775.3 + 775.8 + 775.8 + 776.3}$$			
TSP1/TIVTTLQDSIR	$\frac{623.8}{628.8}$	28 ± 0.3	33 ± 3.7	7
	$$\frac{623.8 + 624.3}{628.8 + 629.3}$$			
RHOB/IQAYDYLECSAK	$\frac{730.8}{734.8}$	20 ± 0.9	15 ± 14.8	12
	$$\frac{730.8 + 731.3}{734.8 + 735.3}$$			

Mean % of heavy peptide deamidations, % of light peptide deamidations and % change in concentration (due to the modified forms) for each peptide, and across all urine samples is shown

We had a single peptide VAGTGEGGLEE**M**VEELNSGK (DBNL) with methionine. This amino acid is particularly susceptible to oxidation as a result of sample storage. Both mono-oxidized and di-oxidized forms have been described. We initially noted a peak in most ADPKD urine samples due to the mono-oxidized form. However, the heavy peptide had no such oxidized forms, although it eluted at a similar retention time. We tried to reverse methionine oxidation by adding 1 µl of 0.3 M methionine solution to 20 µg peptide fractions. This failed to result in the reversal. We then injected two C_18_ microextracted peptide fractions on Q-Exactive mass spectrometer in LC–MS/MS fashion, and could not identify a light spectrum of oxidized or non-oxidized peptide VAGTGEGGLEEMVEELNSGK (albeit in non-fractionated urine samples). We thus abolished monitoring of this peptide.

### Analytical parameters of Ang II-regulated peptides quantified in urine

We next determined the analytical parameters of Ang II-regulated peptides. When heavy and light peptides were monitored, we discovered that peptides VAGTGEGGLEEMVEELNSGK, IFPYEEYASWK, EALCGCSINVPTLDGR, SSGDYSATFLPGLIPAEK, EISDGDVIISGNK, GGGDQGYGSGR could not be confidently identified, due to the absence of co-elution of heavy and light peptides. The remaining seven peptides were confidently identified, and we proceeded to analyze them. We utilized urine samples from two controls and one ADPKD subject to generate calibration curves, and calculate LOD and LOQ for each peptide. Calibration curves were generated from H/L peptide ratios determined by spiking heavy-labeled peptides into aliquots of the same urine sample, at concentrations: 1000 (only for some peptides with higher estimated concentration in urine), 100, 10, 1, 0.1 and 0 fmol/μl. Each point on the calibration curve was generated by duplicate or triplicate injections. Additional file 1: Figure S1 A–G displays calibration curves of the seven monitored peptides. As noted in Additional file 1: Figure S1, all peptides demonstrated excellent linearity with R^2^ > 0.99. LYPLA1 peptide demonstrated linearity with R^2^ > 0.97.

We next analyzed within-run and between-run variability. Table 4 displays mean within-run and between-run variability for each individual peptide in two control and nine ADPKD urine samples. Overall, within-run CV was <10 %. Between-run CV takes into account variation due to sample preparation. We noted higher between-run CVs compared to within-run CVs, although not for peptide LYPLA1 (CV was 6 %). We then explored the reasons for this variability. BSA protein spiked at the very beginning of sample preparation enabled better understanding of the contribution of variability in sample preparation to overall results. As Fig. 2 displays, BSA protein was added at the beginning of sample preparation, at a constant amount, to all urine samples containing the same amount of total protein, while its heavy peptides were added at the very end, just prior to C_18_ microextraction. Any alterations in L/H BSA peptide ratios may be a result of protein loss during sample preparation. Adjustment of peptide concentrations by BSA did not account for variability in all samples or for all peptides, as evident from Additional file 1: Figure S2. For example, samples 1 and 167 still displayed peptide measurements with poor between-run correlation. Also, peptides of GLNA (GLUL) and TSP1 proteins displayed poor between-run correlation in several samples. We thus concluded that adjustment by BSA was not justified, but still used L/H BSA ratio as an indicator of overall sample integrity.

**Table 4:** Peptide analytical parameters including within-run, between-run CV, LOD and LOQ

Protein	Peptide sequence	Within-run CV (%)	Between-run CV (%)	LOD (fmol peptide)	LOD (fmol/µmol Cr)	LOQ (fmol peptide)	LOQ (fmol/µmolCr)
TSP-1	TIVTTLQDSIR	4.6	28	1.1	0.40	1.2	0.43
TSP-1	GGVNDFQGVLQNVR	4.7	32	1.3	0.43	2.3	0.75
GLUL	LVLCEVFK	8.4	16	0.17	0.06	1.1	0.39
RHOB	IQAYDYLECSAK^a^	7.6	24	0.1	0.04	0.1	0.04
BST1	GFFADYEIPNLQK	2.6	32	0.14	0.05	0.48	0.17
LYPLA1	LAGVTALSCWLPLR^a^	4.4	6	0.1	0.04	1.0	0.40
LAMB2	GSCYPATGDLLVGR	3.7	37	1.04	0.34	1.20	0.39

Within-run and between-run CVs are presented as means of 11 urine samples for each peptide

^a^LOD and LOQ could not be determined from statistical methods for this peptide. They were extrapolated from the calibration curve

LOD was calculated as previously described [35, 36] by taking the average of blank injections and adding three standard deviations to the mean. Similarly, LOQ was calculated by taking the average of blank injections and adding 10 standard deviations to the mean. In two cases, LOD and LOQ could not be calculated from linear regression equation. In this case, LOD was estimated as the minimal amount of heavy peptide significantly different from the blank injection. LOQ was estimated as minimal amount of heavy peptide measured within the linear response range of H/L peptide ratio of the calibration curve [37]. Table 4 displays LOD and LOQ for each peptide.

### Quantification of Ang II-regulated peptides in urine samples

In order to quantify Ang II-regulated peptides in relevant urine samples from 11 controls, 17 patients with ADPKD and 9 patients with CKD (Additional file 1: Table S1), we selected three top transitions of each peptide, and ran methods for each sample in duplicates (within-run replicates). The methods are shown in Additional file 1: Table S5. We calculated the mean peptide excretion rate of within-run replicates for each sample and adjusted it by urine creatinine. TSP1 protein was quantified by two different peptides, and urine excretion rates between the two peptides showed excellent linear correlation with R^2^ = 0.86 (Additional file 1: Figure S3). Three samples had LYPA1 levels below LOQ. The number of peptide measurements below LOD was <2 %.

**Fig. 4:**
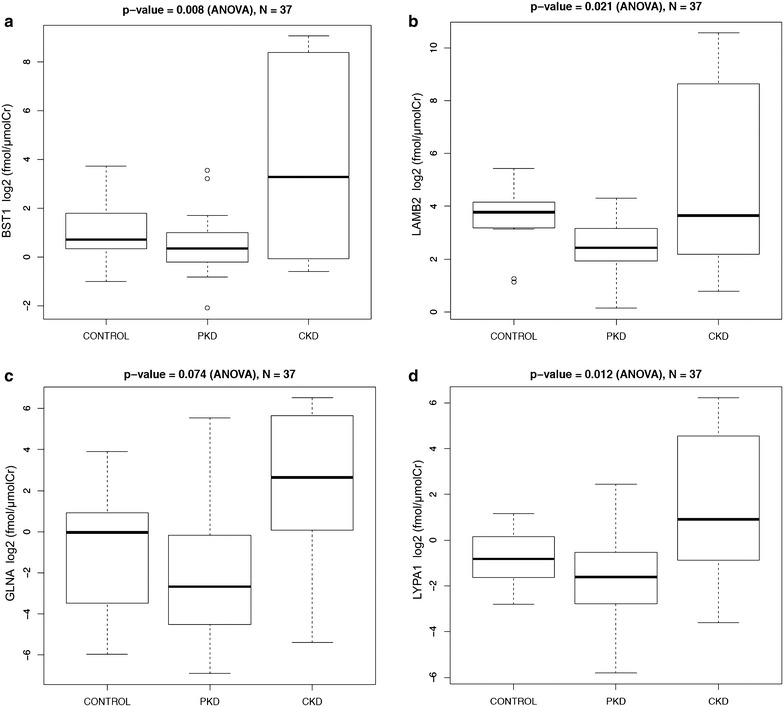
Absolute quantification of Ang II-regulated proteins in urine of healthy controls, patients with polycystic kidney disease (PKD) and chronic kidney disease (CKD). Values are expressed in log2 transformed fmol/µmol of creatinine. **a** BST1, **b** LAMB2, **c** GLNA, **d** LYPA1

**Fig. 5:**
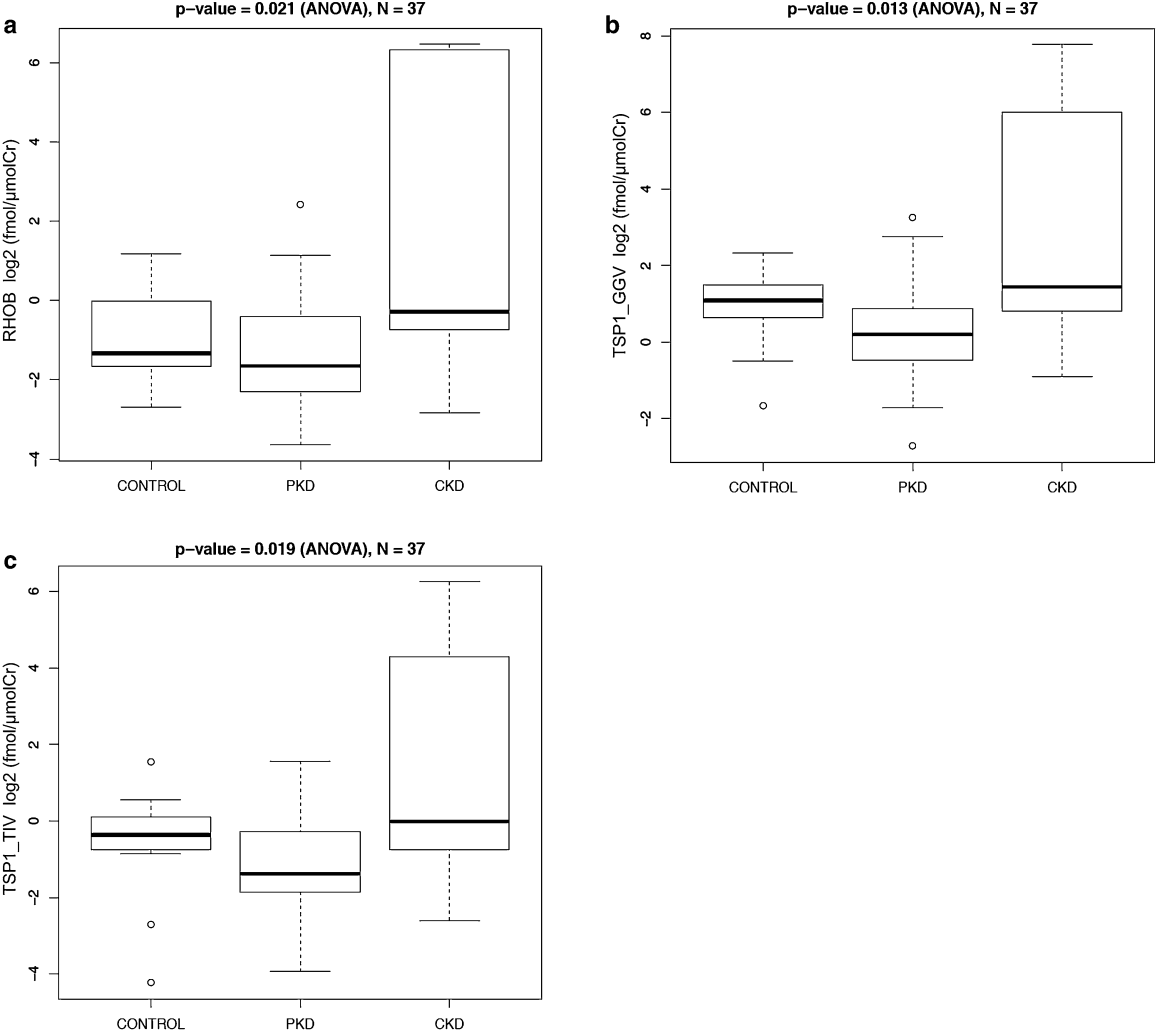
Absolute quantification of Ang II-regulated proteins in urine of healthy controls, patients with polycystic kidney disease (PKD) and chronic kidney disease (CKD). Values are expressed in log2 transformed fmol/µmol of creatinine. **a** RHOB, **b** TSP1—1^st^ peptide and **c** TSP1—2nd peptide

 We found that urine excretion rate of all proteins followed the same pattern, with the lowest excretion rates in patients with ADPKD and the highest excretion rates in patients with CKD (Figs. 4, 5). The urine excretion rate of most proteins was significantly different between the three disease groups: BST1 (p = 0.008, ANOVA), LAMB2 (p = 0.021, ANOVA), LYPA1 (p = 0.012, ANOVA), RHOB (p = 0.021, ANOVA), and TSP1 (p = 0.013 for peptide 1 and p = 0.019 for peptide 2, ANOVA). Post-hoc analysis demonstrated that in all cases there was a statistically significant difference between ADPKD and CKD groups, and in case of BST1, there was also a statistically significant difference between CKD and control groups.

We then examined associations between urine excretion rate of Ang II-regulated proteins and clinical characteristics, such as measures of kidney function including serum creatinine (sCr), total kidney volume, sex, age, and disease group of our patients. There was no correlation between urine excretion rates of Ang II-regulated proteins and sCr, total kidney volume, or age. However, univariate analysis demonstrated significant association between urine excretion rates of all proteins and sex, as well as disease group (Additional file 1: Table S6). In all cases, urine excretion rate of Ang II-regulated proteins was higher in females compared to males. GLNA demonstrated striking association with sex (p = 1.10E−05, two sample t test), but no significant association with disease group (p = 0.074, ANOVA). BST1 demonstrated the strongest association with disease group (p = 0.008, ANOVA).

**Fig. 6:**
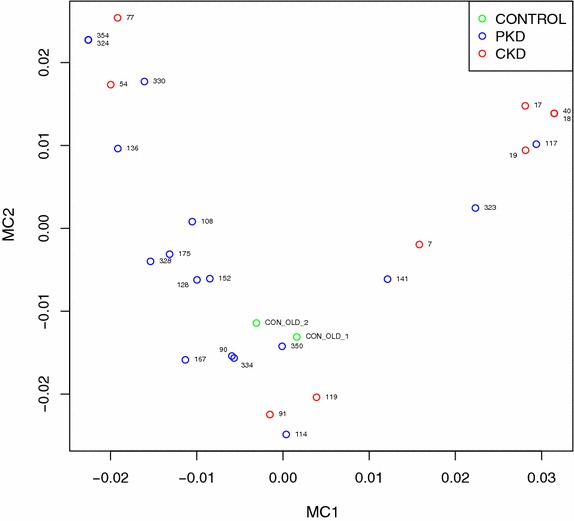
Multivariate analysis using multiple correspondence analysis (MCA) on protein concentration and clinical data including sex and age, where all protein concentrations as well as age were first discretized into four levels each, in a way that preserves equal frequencies between levels. Good separation between CKD and PKD patients is evident

Finally, we performed multivariate analysis using multiple correspondence analysis (MCA) on protein concentration and clinical data including sex and age, where all protein concentrations as well as age were first discretized into four levels each, in a way that preserves equal frequencies between levels (Fig. 6). Only two controls had information about age, so only those could be included. Interestingly, samples 77 and 54 represent CKD patients with unknown etiology of renal disease and without proteinuria or renal dysfunction. Samples 91 and 119 represent patients with medullary sponge kidney, a form of kidney disease with medullary cysts, without proteinuria or renal dysfunction. Finally, the right upper corner contains a cluster of patients with IgA nephropathy, associated with proteinuria and/or impaired kidney function. The two PKD patients 323 and 117 have proteinuria or impaired renal function with large kidneys, suggesting advanced disease. The analysis demonstrates good separation between ADPKD and CKD patients, and thus suggests that urine excretion rate of Ang II-regulated proteins in conjunction with sex and age can distinguish ADPKD and CKD patients.

Lastly, to obtain biological context of the six proteins, we first mapped them to protein interaction network (Additional file 1: Figure S4), and then conducted the pathway enrichment analysis (Additional file 1: Figure S5). Taking the 6 monitored proteins, we have queried IID database to obtain network of immediate, physically interacting proteins. The resulting network comprised 226 proteins and 1575 interactions (Additional file 1: Figure S4). Six query proteins are highlighted as rectangles. None of them directly interact, but all interact via seven proteins—nodes highlighted with red outline, and edges made thicker. While five proteins link only pair of query proteins, ELAV1 links three and 1433E links all six proteins. 1433E is also the most central node in the network (as determined by all pairs shortest path analysis).

## Discussion

We have developed SRM assays for quantification of urine excretion rate of previously discovered Ang II-regulated proteins, in order to address a clinically unmet need, namely, the lack of markers of kidney Ang II activity. Our main objectives were: (1) to develop methods for urine-based quantification of kidney Ang II activity proteins; (2) to optimize peptide quantification in urine samples, and (3) to demonstrate the potential relevance of Ang II-regulated proteins in patients with ADPKD and CKD. These optimized SRM assays have the potential to serve as a clinical tool for assessment of kidney Ang II activity in patients with chronic kidney diseases.

Ang II-regulated proteins were discovered in kidney cells in vitro, and validated in animal models of kidney disease in vivo [26, 27]. The role of RAS and Ang II in the progression of ADPKD and CKD had been established. We first developed SRM-based methods for quantification of Ang II-regulated proteins in human urine. SRM has emerged as an optimal method for specific, multiplexed, and cost-effective quantification of proteins in tissue and biofluids [28, 36–38]. Prior studies utilizing SRM for quantification of urine proteins have been reported [28, 35, 39, 40]. These studies typically applied different methods for protein precipitation, and digestion with two enzymes was not tested. Other studies applied depletion of abundant proteins [41]. The latter approach is suboptimal, as depletion strategies are likely to result in loss of analytes of interest, in addition to adding complexity and increasing the time required for sample processing. The strength of our approach includes: (1) elucidation of an optimal sample preparation method without the need for sample fractionation of depletion of abundant proteins, (2) monitoring of post-translational modifications in urine, and (3) demonstration of potential relevance of these proteins in clinical disease.

We found that combination of digestive enzymes was more critical than the choice of protein concentration strategy. This likely relates to enhanced digestion with multiple enzymes. Furthermore, urine processing is problematic since protein concentration is a necessary step prior to subsequent analyses, given that urine is a dilute biofluid. In order to anticipate the losses associated with urine processing, we introduced purified, full-length BSA protein. This exogenous standard enabled determination of the recovery of BSA protein and thus the integrity of the sample processing. Similar approaches to assessment of yield and reproducibility were applied before [35, 42].

Peptide modifications should be considered when performing quantitative analyses, since peptides may acquire these modifications in unpredictable fashion [43, 44]. These modifications arise as a consequence of protein/peptide aging, or represent artifacts of sample processing [43, 44]. We monitored peptides with glutamine conversion to glutamic acid, and asparagine conversion to aspartic acid. These modifications may serve as ‘molecular clocks’ of protein aging and turnover [45]. We demonstrated increased rates of deamidated endogenous peptides, compared to synthetic ones, and this was previously described [43]. Some of our peptides had more than one potential deamidated site, and we noted that in these cases, single deamidations and N-terminal site modifications tended to predominate. This is not surprising, since peptide amino acid sequence surrounding the glutamine or asparagine residues determines the likelihood of modification [46]. Importantly, heavy peptides appeared to have a stable rate of deamidation, which varied between 20 and 35 %, but was consistent for any given peptide. Light peptides coming from urine samples had more variability in the rate of deamidation. This likely reflects the ‘molecular clock effect’, given that urines came from different individuals, and had been collected at different times. Quantification of deamidated peptide forms is often ignored in SRM studies, and our approach to monitoring then should be applied to other SRM studies that include peptides with potential deamidation sites.

We used BSA protein and the corresponding heavy-labeled peptides to assess different sample processing methods, and similar approaches had been reported previously, using non-human proteins spiked into urine [35, 42]. Variable recovery of BSA protein that we noticed might be explained by the carry-over effect of BSA peptides used as QCs. As such, more ideal internal standards could constitute other non-human proteins with corresponding heavy peptides, or pairs of light and heavy peptides that cover the entire range of hydrophobicities and that are not used in QC. Such proteins and peptides may prove useful when adjusting for any losses during sample processing.

Our last objective was to test the relevance of Ang II-regulated protein measurements in urine of patients with chronic kidney diseases. We quantified Ang II regulated proteins in urine of patients with ADPKD, CKD and healthy controls. Interestingly, all proteins displayed the same direction of change in urine samples, with the highest excretion rate in CKD and the lowest in ADPKD patients. The CKD group consisted mainly of patients with IgA nephropathy, a kidney disease where progression was strongly linked to kidney Ang II activity [47–49]. It is notable that this group of patients had the highest rate of proteinuria (Additional file 1: Table S1), supporting the notion that kidney Ang II was active. It is interesting that ADPKD patients had the lowest levels of Ang II-regulated protein excretion rates. We found that at the level of mRNA, the majority of Ang II-regulated proteins displayed significantly increased expression in kidney cysts compared to normal tissue. Similarly, prior studies of kidney tissue and cysts from patients with polycystic kidney disease demonstrated convincing increases of RAS components, including Ang II [21, 50, 51]. RAS and Ang II activity accelerated cyst growth through production of mitogenic factors and hypertension [52–55]. It is however known that renal cysts could become walled off and lose communication with the tubules. Prior studies support this notion by showing that inflammatory markers highly expressed in ADPKD cysts are excreted in urine at relatively low levels [56]. It is thus possible that our Ang II-regulated proteins had decreased excretion in ADPKD urine because of loss of communication between cysts and the tubules.

We found a strong association between sex and urine excretion rate of Ang II-regulated proteins. It has long been recognized that male sex predisposes to kidney disease progression and that there are differences in RAS activity between the sexes [57–60]. Whether this difference reflects differential protein expression in the kidney is unknown and will be addressed in future studies.

Protein that was most significantly associated with disease groups was BST1. BST1 (bone marrow stromal antigen-1) is an enzyme, ADP-ribosyl cyclase, that converts NAD+ to cyclic ADP ribose, which activates calcium signaling and promotes proliferation in hematopoietic cells [61]. This is somewhat analogous to inositol-1,4,5-triphosphate (IP3), which is produced upon Ang II-mediated signaling, and which releases calcium from the sarcoplasmic reticulum. BST1 is expressed in the normal human kidney, mostly in proximal tubular cells (Human Protein Atlas). However, its function in the kidney is unknown. It is worth mentioning that RHOB and TSP1 were implicated in Ang II-mediated effects. RHOB is involved in Rho GTPase signaling, and was found to be upregulated by Ang II in adrenocortical cells [62]. TSP1 is a profibrotic protein, capable of binding to fibrinogen, fibronectin, collagen and integrins. TSP1 antagonist blocked cardiomyopathy induced by high Ang II in rats [63]. In renal mesangial cells, Ang II-induced activation of latent TGFβ1 was dependent on p38MAPK and JNK signaling via TSP1 [64]. Finally, in a mouse model of unilateral ureteral obstruction, Ang II infusion resulted in intense tubular deposition of TSP1, which was linked to tubulointerstitial fibrosis [65]. TSP1 may thus represent an important cellular signal for Ang II induced fibrosis. It is plausible that quantified proteins participate in the chronic mechanisms of kidney injury that involve fibrosis, proliferation and response to injury, mechanisms linked to Ang II bioactivity. The role of these proteins in ADPKD and CKD should be further explored in the future.

Lastly, the proteins quantified displayed enrichment in the same biological processes and pathways enriched among the original 83 proteins [26], namely profibrotic pathways such as TGFβ, plasminogen and PDGF signaling, Rho protein signal transduction, ERK and MAPK signaling and inflammation (Additional file 1: Figure S5). While none of the 6 proteins interacted directly, they were all connected via 7 proteins (Additional file 1: Figure S4), and protein 1433E connected all 6 Ang II-regulated proteins. 1433E is an adaptor protein implicated in metabolism, PI3K and GPCR signal transduction, apoptosis and cell cycle regulation. All of these processes were significantly enriched by Ang II in kidney cells [1], suggesting that 1433E may in fact link Ang II signaling to all six proteins monitored in urine.

Our study has several strengths, including development of a method for quantification of Ang II-regulated proteins that is readily translatable into the clinic. We have performed careful method optimization, and we monitored modified forms of peptides. We offer a workflow for quantification of urine proteins that can easily be adapted to the study of any urine analyte. Finally, proteins monitored appear to differentiate kidney disease groups and may have relevance in clinical populations.

 Notwithstanding these strengths, there are some limitations. Data regarding age were missing from most healthy controls. Orthogonal measures of Ang II-regulated proteins were not performed. Finally, we have analyzed a small number of samples in this pilot study. The study will be expanded to a larger population of patients with kidney diseases in the future.

## Conclusions

In summary, we have developed methods for SRM-based quantification of Ang II-regulated proteins in human urine, and we demonstrated that urine excretion rates of these proteins carry meaningful information about kidney disease type. Future studies will examine whether these proteins represent coveted markers of kidney Ang II activity in larger cohorts of patients with chronic kidney disease.

## Additional files

10.1186/s12014-016-9117-x **Table S1.** Characteristics of PCKD patients and healthy controls. *Estimated glomerular filtration rate (eGFR) was calculated from MDRD equation or 24-hour urine creatinine excretion rate. ^ψ^Total kidney volume was calculated by magnetic resonance imaging (MRI). **Table S2**. Parameters of a multiplex scheduled SRM assay of all monitored peptides, including peptide sequence, m/z, modifications monitored and charge. **Table S3.** Scheduled SRM parameters of light peptides monitored during method development. **Table S4**. Modified peptides monitored in urine sample 1. Total areas are calculated as the sum of all heavy-labeled and light peptide areas. Total ratios are calculated by dividing the total heavy by total light area for each peptide. **Table S5**. Optimized scheduled SRM methods for heavy-labeled and light peptides. **Table S6**. Associations between urine excretion rate of Ang II-regulated proteins and clinical characteristics such as sex, age and disease group. Numbers represent p-values. For variable sex, two sample t-test was performed. For age, t-test for correlation testing was performed. For disease groups, ANOVA was used. *p<0.05 **p<0.01***p<0.001. Additional file 1: **Figure S1.** Calibration curves for 7 Ang II-regulated peptides. A) TSP1 peptide TIVTTLQDSIR. y = -0.2239 + 0.2930 x; R^2^ = 0.9997; B) TSP1 peptide GGVNDFQGVLQNVR. y = 0.06662 + 0.1242 x; R^2^ = 0.999; C) GLUL peptide. y = 0.08380 + 0.09353 x; R^2^= 0.9969; D) RHOB peptide. y = 0.3098 + 0.2902 x; R^2^ = 0.9935; E) BST1 peptide. y = 0.05329 + 0.03982x; R^2^= 0.9995; F) LYPLA1 peptide. y = 0.2281 + 0.1405 x; R^2^ = 0.9794; G) LAMB2 peptide. y = -0.2221 + 0.2719 x; R^2^ = 0.9998. Values at the low range of the curve are shown on the top left. **Figure S2**. Between-run variability of L/H peptide ratios adjusted for L/H BSA ratios in different **A)** urine samples (each box represents one urine sample) and **B)** peptides (each box represents one peptide). X-axis represents log2-transformed adjusted L/H ratios in urine sample A, divided by the L/H ratio in urine sample B. Samples A and B represent aliquots of the same urine sample that were thawed and processed on different days. Log2-transformed ratios thus represent a measure of between-run variability, and perfectly reproducible measurements between samples A and B would have a log2-transformed ratio of 0. **Figure S3.** Linear correlation between urine excretion rates of two peptides of TSP1. Peptide 1 measurements are presented on x-axis, while peptide 2 measurements are on y-axis. **Figure S4.** Protein-protein interaction network including the 6 monitored proteins and their direct interaction partners and interaction among them. Physical protein interactions obtained from IID v2016-03 (26516188), and visualization performed in NAViGaTOR 2.3 (19837718). Query proteins are highlighted as rectangles. Nodes with red signify the shortest path connection among query proteins. Node color represent biological function, as per legend. All other nodes and edges are partially transparent to reduce network clutter. Corresponding XML file can be accessed at: http://www.cs.utoronto.ca/~juris/data/ClinProteom16/. **Figure S5.** Pathway enrichment analysis among the 6 monitored proteins and their PPI partners from Figure S4 using pathDIP ver. 1.0. Only top 60 most significantly enriched pathways taken from sources listed to the right of the graph are displayed. Note, p-values representing significance of some of these pathways were equal to zero, to allow their log10 transformation for visualization purposes, those were adjusted to 10^-18^.
10.1186/s12014-016-9117-x Data file used for analyses described in the manuscript.

## Abbreviations

ACE: angiotensin converting enzyme
ADPKD: autosomal dominant polycystic kidney disease
Ang II: angiotensin II
BLAST: basic local alignment search tool
BSA: bovine serum albumin
BST1: bone marrow stromal cell antigen 1
CKD: chronic kidney disease
CV: coefficient of variation
eGFR: estimated glomerular filtration rate
FDR: false detection rate
LAMB2: laminin beta 2
LC–MS: liquid chromatography–mass spectrometry
LOD: limit of detection
LOQ: limit of quantification
LYPA1: lysophospholipase I
MRI: magnetic resonance imaging
RAS: renin angiotensin system
RHOB: ras homolog family member B
SAM: significance analysis of microarrays
SD: standard deviation
SRM: selected reaction monitoring
TSP1: thrombospondin-1

## References

[1] Zimmerman JB, Robertson D, Jackson EK (1987). Angiotensin II-noradrenergic interactions in renovascular hypertensive rats. J Clin Invest.

[2] Purdy RE, Weber MA (1988). Angiotensin II amplification of alpha-adrenergic vasoconstriction: role of receptor reserve. Circ Res.

[3] Wolf G, Ziyadeh FN, Zahner G, Stahl RA (1995). Angiotensin II-stimulated expression of transforming growth factor beta in renal proximal tubular cells: attenuation after stable transfection with the c-mas oncogene. Kidney Int.

[4] Wolf G, Wenzel U, Hannken T, Stahl RA (2001). Angiotensin II induces p27(Kip1) expression in renal tubules in vivo: role of reactive oxygen species. J Mol Med (Berl).

[5] Wolf G, Zahner G, Schroeder R, Stahl RA (1996). Transforming growth factor beta mediates the angiotensin-II-induced stimulation of collagen type IV synthesis in cultured murine proximal tubular cells. Nephrol Dial Transplant.

[6] Sanz AB, Sanchez-Nino MD, Ramos AM, Moreno JA, Santamaria B, Ruiz-Ortega M, Egido J, Ortiz A (2010). NF-kappaB in renal inflammation. J Am Soc Nephrol.

[7] Crowley SD, Frey CW, Gould SK, Griffiths R, Ruiz P, Burchette JL, Howell DN, Makhanova N, Yan M, Kim HS (2008). Stimulation of lymphocyte responses by angiotensin II promotes kidney injury in hypertension. Am J Physiol Renal Physiol.

[8] Schrier RW (2009). Renal volume, renin-angiotensin-aldosterone system, hypertension, and left ventricular hypertrophy in patients with autosomal dominant polycystic kidney disease. J Am Soc Nephrol.

[9] Gabow PA, Chapman AB, Johnson AM, Tangel DJ, Duley IT, Kaehny WD, Manco-Johnson M, Schrier RW (1990). Renal structure and hypertension in autosomal dominant polycystic kidney disease. Kidney Int.

[10] Grantham JJ, Torres VE, Chapman AB, Guay-Woodford LM, Bae KT, King BF, Wetzel LH, Baumgarten DA, Kenney PJ, Harris PC (2006). Volume progression in polycystic kidney disease. N Engl J Med.

[11] Bae K, Park B, Sun H, Wang J, Tao C, Chapman AB, Torres VE, Grantham JJ, Mrug M, Bennett WM (2013). Segmentation of individual renal cysts from MR images in patients with autosomal dominant polycystic kidney disease. Clin J Am Soc Nephrol.

[12] Schrier R, McFann K, Johnson A, Chapman A, Edelstein C, Brosnahan G, Ecder T, Tison L (2002). Cardiac and renal effects of standard versus rigorous blood pressure control in autosomal-dominant polycystic kidney disease: results of a seven-year prospective randomized study. J Am Soc Nephrol.

[13] Ecder T, Edelstein CL, Fick-Brosnahan GM, Johnson AM, Chapman AB, Gabow PA, Schrier RW (2001). Diuretics versus angiotensin-converting enzyme inhibitors in autosomal dominant polycystic kidney disease. Am J Nephrol.

[14] Schrier RW, McFann KK, Johnson AM (2003). Epidemiological study of kidney survival in autosomal dominant polycystic kidney disease. Kidney Int.

[15] Jafar TH, Stark PC, Schmid CH, Strandgaard S, Kamper AL, Maschio G, Becker G, Perrone RD, Levey AS (2005). The effect of angiotensin-converting-enzyme inhibitors on progression of advanced polycystic kidney disease. Kidney Int.

[16] Mann JF, Schmieder RE, McQueen M, Dyal L, Schumacher H, Pogue J, Wang X, Maggioni A, Budaj A, Chaithiraphan S (2008). Renal outcomes with telmisartan, ramipril, or both, in people at high vascular risk (the ONTARGET study): a multicentre, randomised, double-blind, controlled trial. Lancet.

[17] Fried LF, Emanuele N, Zhang JH, Brophy M, Conner TA, Duckworth W, Leehey DJ, McCullough PA, O’Connor T, Palevsky PM (2013). Combined angiotensin inhibition for the treatment of diabetic nephropathy. N Engl J Med.

[18] Vos PF, Boer P, Braam B, Koomans HA (1994). The origin of urinary angiotensins in humans. J Am Soc Nephrol.

[19] Yamamoto T, Nakagawa T, Suzuki H, Ohashi N, Fukasawa H, Fujigaki Y, Kato A, Nakamura Y, Suzuki F, Hishida A (2007). Urinary angiotensinogen as a marker of intrarenal angiotensin II activity associated with deterioration of renal function in patients with chronic kidney disease. J Am Soc Nephrol.

[20] Mills KT, Kobori H, Hamm LL, Alper AB, Khan IE, Rahman M, Navar LG, Liu Y, Browne GM, Batuman V (2012). Increased urinary excretion of angiotensinogen is associated with risk of chronic kidney disease. Nephrol Dial Transplant.

[21] Park HC, Kang AY, Jang JY, Kim H, Han M, Oh KH, Kim SH, Noh JW, Cheong HI, Hwang YH, Ahn C (2015). Increased urinary Angiotensinogen/Creatinine (AGT/Cr) ratio may be associated with reduced renal function in autosomal dominant polycystic kidney disease patients. BMC Nephrol.

[22] Kocyigit I, Yilmaz MI, Unal A, Ozturk F, Eroglu E, Yazici C, Orscelik O, Sipahioglu MH, Tokgoz B, Oymak O (2013). A link between the intrarenal renin angiotensin system and hypertension in autosomal dominant polycystic kidney disease. Am J Nephrol.

[23] Lifton RP (1995). Genetic determinants of human hypertension. Proc Natl Acad Sci USA.

[24] Matsusaka T, Niimura F, Shimizu A, Pastan I, Saito A, Kobori H, Nishiyama A, Ichikawa I (2012). Liver angiotensinogen is the primary source of renal angiotensin II. J Am Soc Nephrol.

[25] Matsusaka T, Niimura F, Pastan I, Shintani A, Nishiyama A, Ichikawa I (2014). Podocyte injury enhances filtration of liver-derived angiotensinogen and renal angiotensin II generation. Kidney Int.

[26] Konvalinka A, Zhou J, Dimitromanolakis A, Drabovich AP, Fang F, Gurley S, Coffman T, John R, Zhang SL, Diamandis EP, Scholey JW (2013). Determination of an angiotensin II-regulated proteome in primary human kidney cells by stable isotope labeling of amino acids in cell culture (SILAC). J Biol Chem.

[27] Bae EH, Konvalinka A, Fang F, Zhou X, Williams V, Maksimowski N, Song X, Zhang SL, John R, Oudit GY (2015). Characterization of the intrarenal renin-angiotensin system in experimental alport syndrome. Am J Pathol.

[28] Soste M, Selevsek N, Rost H, Sethi A, Carapito C, Farrah T, Deutsch EW, Kusebauch U, Moritz RL (2012). Reproducible quantification of cancer-associated proteins in body fluids using targeted proteomics. Sci Transl Med.

[29] Drabovich AP, Pavlou MP, Dimitromanolakis A, Diamandis EP (2012). Quantitative analysis of energy metabolic pathways in MCF-7 breast cancer cells by selected reaction monitoring assay. Mol Cell Proteomics.

[30] Drabovich AP, Jarvi K, Diamandis EP (2011). Verification of male infertility biomarkers in seminal plasma by multiplex selected reaction monitoring assay. Mol Cell Proteomics.

[31] Drabovich AP, Diamandis EP (2010). Combinatorial peptide libraries facilitate development of multiple reaction monitoring assays for low-abundance proteins. J Proteome Res.

[32] Maclean B, Tomazela DM, Abbatiello SE, Zhang S, Whiteaker JR, Paulovich AG, Carr SA, Maccoss MJ (2010). Effect of collision energy optimization on the measurement of peptides by selected reaction monitoring (SRM) mass spectrometry. Anal Chem.

[33] MacLean B, Tomazela DM, Shulman N, Chambers M, Finney GL, Frewen B, Kern R, Tabb DL, Liebler DC, MacCoss MJ (2010). Skyline: an open source document editor for creating and analyzing targeted proteomics experiments. Bioinformatics.

[34] Song X, Di Giovanni V, He N, Wang K, Ingram A, Rosenblum ND, Pei Y (2009). Systems biology of autosomal dominant polycystic kidney disease (ADPKD): computational identification of gene expression pathways and integrated regulatory networks. Hum Mol Genet.

[35] Selevsek N, Matondo M, Sanchez Carbayo M, Aebersold R, Domon B (2011). Systematic quantification of peptides/proteins in urine using selected reaction monitoring. Proteomics.

[36] Kennedy JJ, Abbatiello SE, Kim K, Yan P, Whiteaker JR, Lin C, Kim JS, Zhang Y, Wang X, Ivey RG (2014). Demonstrating the feasibility of large-scale development of standardized assays to quantify human proteins. Nat Methods.

[37] Drabovich AP, Dimitromanolakis A, Saraon P, Soosaipillai A, Batruch I, Mullen B, Jarvi K, Diamandis EP (2013). Differential diagnosis of azoospermia with proteomic biomarkers ECM1 and TEX101 quantified in seminal plasma. Sci Transl Med.

[38] Picotti P, Aebersold R (2012). Selected reaction monitoring-based proteomics: workflows, potential, pitfalls and future directions. Nat Methods.

[39] Shi T, Gao Y, Quek SI, Fillmore TL, Nicora CD, Su D, Zhao R, Kagan J, Srivastava S, Rodland KD (2014). A highly sensitive targeted mass spectrometric assay for quantification of AGR2 protein in human urine and serum. J Proteome Res.

[40] Gallien S, Duriez E, Crone C, Kellmann M, Moehring T, Domon B (2012). Targeted proteomic quantification on quadrupole-orbitrap mass spectrometer. Mol Cell Proteomics.

[41] Craciun FL, Bijol V, Ajay AK, Rao P, Kumar RK, Hutchinson J, Hofmann O, Joshi N, Luyendyk JP, Kusebauch U (2016). RNA sequencing identifies novel translational biomarkers of kidney fibrosis. J Am Soc Nephrol.

[42] Court M, Selevsek N, Matondo M, Allory Y, Garin J, Masselon CD, Domon B (2011). Toward a standardized urine proteome analysis methodology. Proteomics.

[43] Martinez-Morillo E, Nielsen HM, Batruch I, Drabovich AP, Begcevic I, Lopez MF, Minthon L, Bu G, Mattsson N, Portelius E (2014). Assessment of peptide chemical modifications on the development of an accurate and precise multiplex selected reaction monitoring assay for apolipoprotein e isoforms. J Proteome Res.

[44] Lange V, Picotti P, Domon B, Aebersold R (2008). Selected reaction monitoring for quantitative proteomics: a tutorial. Mol Syst Biol.

[45] Robinson NE, Robinson AB (2001). Molecular clocks. Proc Natl Acad Sci USA.

[46] Bischoff R, Kolbe HV (1994). Deamidation of asparagine and glutamine residues in proteins and peptides: structural determinants and analytical methodology. J Chromatogr B Biomed Appl.

[47] Cattran DC, Greenwood C, Ritchie S (1994). Long-term benefits of angiotensin-converting enzyme inhibitor therapy in patients with severe immunoglobulin a nephropathy: a comparison to patients receiving treatment with other antihypertensive agents and to patients receiving no therapy. Am J Kidney Dis.

[48] Praga M, Gutierrez E, Gonzalez E, Morales E, Hernandez E (2003). Treatment of IgA nephropathy with ACE inhibitors: a randomized and controlled trial. J Am Soc Nephrol.

[49] Li PK, Leung CB, Chow KM, Cheng YL, Fung SK, Mak SK, Tang AW, Wong TY, Yung CY, Yung JC (2006). Hong Kong study using valsartan in IgA nephropathy (HKVIN): a double-blind, randomized, placebo-controlled study. Am J Kidney Dis.

[50] Graham PC, Lindop GB (1988). The anatomy of the renin-secreting cell in adult polycystic kidney disease. Kidney Int.

[51] Torres VE, Donovan KA, Scicli G, Holley KE, Thibodeau SN, Carretero OA, Inagami T, McAteer JA, Johnson CM (1992). Synthesis of renin by tubulocystic epithelium in autosomal-dominant polycystic kidney disease. Kidney Int.

[52] Watanabe G, Lee RJ, Albanese C, Rainey WE, Batlle D, Pestell RG (1996). Angiotensin II activation of cyclin D1-dependent kinase activity. J Biol Chem.

[53] Thomas W, Dooley R, Harvey BJ (2010). Aldosterone as a renal growth factor. Steroids.

[54] Orskov B, Sorensen VR, Feldt-Rasmussen B, Strandgaard S (2012). Changes in causes of death and risk of cancer in Danish patients with autosomal dominant polycystic kidney disease and end-stage renal disease. Nephrol Dial Transplant.

[55] Gabow PA, Johnson AM, Kaehny WD, Kimberling WJ, Lezotte DC, Duley IT, Jones RH (1992). Factors affecting the progression of renal disease in autosomal-dominant polycystic kidney disease. Kidney Int.

[56] Parikh CR, Dahl NK, Chapman AB, Bost JE, Edelstein CL, Comer DM, Zeltner R, Tian X, Grantham JJ, Somlo S (2012). Evaluation of urine biomarkers of kidney injury in polycystic kidney disease. Kidney Int.

[57] Neugarten J, Acharya A, Silbiger SR (2000). Effect of gender on the progression of nondiabetic renal disease: a meta-analysis. J Am Soc Nephrol.

[58] Yamaleyeva LM, Gilliam-Davis S, Almeida I, Brosnihan KB, Lindsey SH, Chappell MC (2012). Differential regulation of circulating and renal ACE2 and ACE in hypertensive mRen2.Lewis rats with early-onset diabetes. Am J Physiol Renal Physiol.

[59] de Alencar Franco Costa D, Todiras M, Campos LA, Cipolla-Neto J, Bader M, Baltatu OC (2015). Sex-dependent differences in renal angiotensinogen as an early marker of diabetic nephropathy. Acta Physiol (Oxf).

[60] Neugarten J, Golestaneh L (2013). Gender and the prevalence and progression of renal disease. Adv Chronic Kidney Dis.

[61] Podesta M, Benvenuto F, Pitto A, Figari O, Bacigalupo A, Bruzzone S, Guida L, Franco L, Paleari L, Bodrato N (2005). Concentrative uptake of cyclic ADP-ribose generated by BST-1 + stroma stimulates proliferation of human hematopoietic progenitors. J Biol Chem.

[62] Romero DG, Plonczynski M, Vergara GR, Gomez-Sanchez EP, Gomez-Sanchez CE (2004). Angiotensin II early regulated genes in H295R human adrenocortical cells. Physiol Genomics.

[63] Belmadani S, Bernal J, Wei CC, Pallero MA, Dell’italia L, Murphy-Ullrich JE, Berecek KH (2007). A thrombospondin-1 antagonist of transforming growth factor-beta activation blocks cardiomyopathy in rats with diabetes and elevated angiotensin II. Am J Pathol.

[64] Naito T, Masaki T, Nikolic-Paterson DJ, Tanji C, Yorioka N, Kohno N (2004). Angiotensin II induces thrombospondin-1 production in human mesangial cells via p38 MAPK and JNK: a mechanism for activation of latent TGF-beta1. Am J Physiol Renal Physiol.

[65] Ma LJ, Yang H, Gaspert A, Carlesso G, Barty MM, Davidson JM, Sheppard D, Fogo AB (2003). Transforming growth factor-beta-dependent and -independent pathways of induction of tubulointerstitial fibrosis in beta6(−/−) mice. Am J Pathol.

