# Supplementary material for: Quantification of angiotensin II-regulated proteins in urine of patients with polycystic and other chronic kidney diseases by selected reaction monitoring
Source: Clin Proteomics. 2016 Aug 5;13:16. doi: 10.1186/s12014-016-9117-x (PMC4974759; doi:10.1186/s12014-016-9117-x)

**Table S1.** Characteristics of patients and healthy controls. *Estimated glomerular filtration rate (eGFR) was calculated from MDRD equation. ^ψ^Total kidney volume was calculated by magnetic resonance imaging (MRI).

| Patient groups | Healthy Controls  (n = 11) | ADPKD  (n = 17) | CKD  (n = 9) |
| --- | --- | --- | --- |
| Age (mean±SD) | 37.5 ± 0.7 | 43.9 ± 16.6 | 47.4 ± 17.2 |
| Sex (F/M) | 6/5 | 7/10 | 5/4 |
| Total kidney volume in mL (mean±SD) ^ψ^ | NA | 1364 ± 1109 | NA |
| Serum Creatinine (μmol/L) (mean±SD) | NA | 100.8 ± 46.7 | 95.0 ± 48.2 |
| eGFR (ml/min) (mean±SD)* | NA | 74.5 ± 28.6 | 75.88 ± 28.8 |
| Urine protein-to-creatinine ratio (mg/mmolCr)  (mean±SD) | 7.9 ± 3.2 | 9.3 ± 5.9 | 71.3 ± 100.8 |

**Table S2**. Parameters of a multiplex scheduled SRM assay of all monitored peptides, including peptide sequence, m/z, modifications monitored and charge.

| Protein | Peptide sequence | m/z | | Modifications | Charge | |
| --- | --- | --- | --- | --- | --- | --- |
| HMOX1 | TEPELLVAHAYTR | | 750.396 | None | | 2 |
| HMOX1 | TEPELLVAHAYTR[+10] | | 755.400 | Heavy R | | 2 |
| HMOX1 | VQDSAPVETPR | | 599.807 | None | | 2 |
| HMOX1 | VQDSAPVETPR[+10] | | 604.811 | Heavy R | | 2 |
| HMOX1 | VQ[+1.0]DSAPVETPR | | 600.299 | Deamidated Q | | 2 |
| HMOX1 | VQ[+1.0]DSAPVETPR[+10] | | 605.303 | Deamidated Q; heavy R | | 2 |
| THBS1 | GGVNDNFQGVLQNVR | | 808.911 | None | | 2 |
| THBS1 | GGVN[+1]DNFQGVLQNVR | | 809.403 | Deamidated N | | 2 |
| THBS1 | GGVNDN[+1]FQGVLQNVR | | 809.403 | Deamidated N | | 2 |
| THBS1 | GGVNDNFQGVLQN[+1]VR | | 809.403 | Deamidated N | | 2 |
| THBS1 | GGVNDNFQGVLQ[+1]NVR | | 809.403 | Deamidated Q | | 2 |
| THBS1 | GGVNDNFQ[+1]GVLQNVR | | 809.403 | Deamidated Q | | 2 |
| THBS1 | GGVNDNFQ[+1.0]GVLQ[+1.0]NVR | | 809.895 | Deamidated Q | | 2 |
| THBS1 | GGVN[+1.0]DN[+1.0]FQGVLQN[+1.0]VR | | 810.387 | Deamidated Q; deamidated N | | 2 |
| THBS1 | GGVN[+1.0]DN[+1.0]FQ[+1.0]GVLQ[+1.0]NVR | | 810.879 | Deamidated Q; deamidated N | | 2 |
| THBS1 | GGVN[+1.0]DN[+1.0]FQ[+1.0]GVLQ[+1.0]N[+1.0]VR | | 811.371 | Deamidated Q; deamidated N | | 2 |
| THBS1 | GGVNDNFQGVLQNVR[+10] | | 813.915 | Heavy R | | 2 |
| THBS1 | GGVN[+1]DNFQGVLQNVR[+10] | | 814.407 | Deamidated N; Heavy R | | 2 |
| THBS1 | GGVNDN[+1]FQGVLQNVR[+10] | | 814.407 | Deamidated N; Heavy R | | 2 |
| THBS1 | GGVNDNFQGVLQN[+1]VR[+10] | | 814.407 | Deamidated N; Heavy R | | 2 |
| THBS1 | GGVNDNFQGVLQ[+1]NVR[+10] | | 814.407 | Deamidated Q; Heavy R | | 2 |
| THBS1 | GGVNDNFQ[+1]GVLQNVR[+10] | | 814.407 | Deamidated Q; Heavy R | | 2 |
| THBS1 | GGVNDNFQ[+1.0]GVLQ[+1.0]NVR[+10] | | 814.899 | Deamidated Q; Heavy R | | 2 |
| THBS1 | GGVN[+1.0]DN[+1.0]FQ[+1.0]GVLQ[+1.0]NVR[+10] | | 815.883 | Deamidated Q; deamidated N; Heavy R | | 2 |
| THBS1 | GGVN[+1.0]DN[+1.0]FQGVLQN[+1.0]VR[+10] | | 815.391 | Deamidated Q; deamidated N; Heavy R | | 2 |
| THBS1 | GGVN[+1.0]DN[+1.0]FQ[+1.0]GVLQ[+1.0]N[+1.0]VR[+10] | | 816.375 | Deamidated Q; deamidated N; Heavy R | | 2 |
| THBS1 | TIVTTLQDSIR | | 623.854 | None | | 2 |
| THBS1 | TIVTTLQ[+1.0]DSIR | | 624.346 | Deamidated Q | | 2 |
| THBS1 | TIVTTLQDSIR[+10] | | 628.858 | Heavy R | | 2 |
| THBS1 | TIVTTLQ[+1.0]DSIR[+10] | | 629.350 | Deamidated Q; Heavy R | | 2 |
| PDCD4 | SGVPVLAVSLALEGK | | 720.427 | None | | 2 |
| PDCD4 | APQLVGQFIAR | | 600.348 | None | | 2 |
| PDCD4 | APQ[+1.0]LVGQFIAR | | 600.840 | Deamidated Q | | 2 |
| PDCD4 | APQ[+1.0]LVGQ[+1.0]FIAR | | 601.332 | Deamidated Q | | 2 |
| PHLA1 | AAGNGEAEPSGGPSYAGR | | 824.372 | None | | 2 |
| PHLA1 | AAGN[+1.0]GEAEPSGGPSYAGR | | 824.864 | Deamidated N | | 2 |
| ARHG2 | ELLSNVDEGIYQLEK | | 875.449 | None | | 2 |
| ARHG2 | ELLSN[+1.0]VDEGIYQLEK | | 875.941 | Deamidated N | | 2 |
| ARHG2 | ELLSN[+1.0]VDEGIYQ[+1.0]LEK | | 876.433 | Deamidated Q; deamidated N | | 2 |
| ARHG2 | DLLVGPGVELLLTPR | | 796.475 | None | | 2 |
| VCPIP1 | SSGDYSATFLPGLIPAEK | | 926.970 | None | | 2 |
| VCPIP1 | SSGDYSATFLPGLIPAEK[+8] | | 930.977 | Heavy K | | 2 |
| VCPIP1 | TEPSVFTASSSNSELIR | | 912.952 | None | | 2 |
| VCPIP1 | TEPSVFTASSSN[+1.0]SELIR | | 913.444 | Deamidated N | | 2 |
| SPARC | YIPPC[+57.0]LDSELTEFPLR | | 975.488 | Carbamidomethylated C | | 2 |
| SPARC | FFETC[+57.0]DLDNDK | | 702.293 | Carbamidomethylated C | | 2 |
| SPARC | FFETC[+57.0]DLDN[+1.0]DK | | 702.785 | Carbamidomethylated C; deamidated N | | 2 |
| GLUL | LVLC[+57.0]EVFK | | 504.283 | Carbamidomethylated C | | 2 |
| GLUL | LVLC[+57.0]EVFK[+8] | | 508.290 | Carbamidomethylated C; Heavy K | | 2 |
| GLUL | TC[+57.0]LLNETGDEPFQYK | | 907.917 | Carbamidomethylated C | | 2 |
| GLUL | TC[+57.0]LLN[+1.0]ETGDEPFQYK | | 908.409 | Carbamidomethylated C; deamidated N | | 2 |
| GLUL | TC[+57.0]LLN[+1.0]ETGDEPFQ[+1.0]YK | | 908.901 | Carbamidomethylated C; deamidated N; deamidated Q | | 2 |
| GLUL | LTGFHETSNINDFSAGVANR | | 1075.517 | None | | 2 |
| GLUL | LTGFHETSN[+1.0]INDFSAGVANR | | 1076.009 | Deamidated N | | 2 |
| GLUL | LTGFHETSN[+1.0]INDFSAGVAN[+1.0]R | | 1076.501 | Deamidated N | | 2 |
| GLUL | LTGFHETSN[+1.0]IN[+1.0]DFSAGVANR | | 1076.501 | Deamidated N | | 2 |
| GLUL | LTGFHETSN[+1.0]IN[+1.0]DFSAGVAN[+1.0]R | | 1076.993 | Deamidated N | | 2 |
| TGFR2 | IFPYEEYASWK | | 716.843 | None | | 2 |
| TGFR2 | IFPYEEYASWK[+8] | | 720.850 | Heavy K | | 2 |
| TGFR2 | LDPTLSVDDLANSGQVGTAR | | 1015.013 | None | | 2 |
| TGFR2 | LDPTLSVDDLANSGQ[+1.0]VGTAR | | 1015.505 | Deamidated Q | | 2 |
| TGFR2 | LDPTLSVDDLAN[+1.0]SGQ[+1.0]VGTAR | | 1015.997 | Deamidated Q; deamidated N | | 2 |
| BST1 | GFFADYEIPNLQK | | 771.385 | None | | 2 |
| BST1 | GFFADYEIPN[+1.0]LQK | | 771.877 | Deamidated N | | 2 |
| BST1 | GFFADYEIPNLQ[+1.0]K | | 771.877 | Deamidated Q | | 2 |
| BST1 | GFFADYEIPN[+1.0]LQ[+1.0]K | | 772.369 | Deamidated Q; deamidated N | | 2 |
| BST1 | GFFADYEIPNLQK[+8] | | 775.392 | Heavy K | | 2 |
| BST1 | GFFADYEIPN[+1.0]LQK[+8] | | 775.884 | Deamidated N; Heavy K | | 2 |
| BST1 | GFFADYEIPNLQ[+1.0]K[+8] | | 775.884 | Deamidated Q; Heavy K | | 2 |
| BST1 | GFFADYEIPN[+1.0]LQ[+1.0]K[+8] | | 776.376 | Deamidated Q; deamidated N; Heavy K | | 2 |
| BST1 | AGLIIPLFLVLASR | | 741.974 | None | | 2 |
| LYPLA1 | LAGVTALSC[+57.0]WLPLR | | 778.935 | Carbamidomethylated C | | 2 |
| LYPLA1 | LAGVTALSC[+57.0]WLPLR[+10] | | 783.939 | Carbamidomethylated C; Heavy R | | 2 |
| LYPLA1 | ASFPQGPIGGANR | | 636.328 | None | | 2 |
| LYPLA1 | ASFPQ[+1.0]GPIGGANR | | 636.820 | Deamidated Q | | 2 |
| LYPLA1 | ASFPQ[+1.0]GPIGGAN[+1.0]R | | 637.312 | Deamidated Q; deamidated N | | 2 |
| LAMB2 | GSC[+57.0]YPATGDLLVGR | | 733.359 | Carbamidomethylated C | | 2 |
| LAMB2 | GSC[+57.0]YPATGDLLVGR[+10] | | 738.363 | Carbamidomethylated C; Heavy R | | 2 |
| LAMB2 | LQEGQTLEFLVASVPK | | 879.985 | None | | 2 |
| LAMB2 | LQ[+1.0]EGQTLEFLVASVPK | | 880.477 | Deamidated Q | | 2 |
| LAMB2 | LQ[+1.0]EGQ[+1.0]TLEFLVASVPK | | 880.969 | Deamidated Q | | 2 |
| EGFR | EISDGDVIISGNK | | 673.844 | None | | 2 |
| EGFR | EISDGDVIISGN[+1.0]K | | 674.336 | Deamidated N | | 2 |
| EGFR | EISDGDVIISGNK[+8] | | 677.851 | Heavy K | | 2 |
| EGFR | EISDGDVIISGN[+1.0]K[+8] | | 678.343 | Deamidated N; Heavy K | | 2 |
| EGFR | ELVEPLTPSGEAPNQALLR | | 1017.547 | None | | 2 |
| EGFR | ELVEPLTPSGEAPN[+1.0]QALLR | | 1018.039 | Deamidated N | | 2 |
| EGFR | ELVEPLTPSGEAPN[+1.0]Q[+1.0]ALLR | | 1018.531 | Deamidated Q; deamidated N | | 2 |
| RBM3 | GGGDQGYGSGR | | 505.718 | None | | 2 |
| RBM3 | GGGDQ[+1.0]GYGSGR | | 506.210 | Deamidated Q | | 2 |
| RBM3 | GGGDQGYGSGR[+10] | | 510.722 | Heavy R | | 2 |
| RBM3 | GGGDQ[+1.0]GYGSGR[+10] | | 511.214 | Deamidated Q; Heavy R | | 2 |
| RBM3 | YYDSRPGGYGYGYGR | | 865.882 | None | | 2 |
| RHOB | LVVVGDGAC[+57.0]GK | | 537.784 | None | | 2 |
| RHOB | IQAYDYLEC[+57.0]SAK | | 730.840 | None | | 2 |
| RHOB | IQ[+1.0]AYDYLEC[+57.0]SAK | | 731.332 | Deamidated Q | | 2 |
| RHOB | IQAYDYLEC[+57.0]SAK[+8] | | 734.847 | Heavy K | | 2 |
| RHOB | IQ[+1.0]AYDYLEC[+57.0]SAK[+8] | | 735.339 | Deamidated Q; Heavy K | | 2 |
| RHOB | EVFETATR | | 476.740 | None | | 2 |
| DBNL | VAGTGEGGLEEMVEELNSGK | | 1003.473 | None | | 2 |
| DBNL | VAGTGEGGLEEM[+16.0]VEELNSGK | | 1011.470 | Oxidized M | | 2 |
| DBNL | VAGTGEGGLEEMVEELN[+1.0]SGK | | 1003.965 | Deamidated N | | 2 |
| DBNL | VAGTGEGGLEEM[+16.0]VEELN[+1.0]SGK | | 1011.962 | Oxidized M; deamidated N | | 2 |
| DBNL | VAGTGEGGLEEM[+32.0]VEELNSGK | | 1019.468 | Dioxidized M | | 2 |
| DBNL | VAGTGEGGLEEM[+32.0]VEELN[+1.0]SGK | | 1019.960 | Dioxidized M; deamidated N | | 2 |
| DBNL | VAGTGEGGLEEMVEELNSGK[+8] | | 1007.480 | Heavy K | | 2 |
| DBNL | VAGTGEGGLEEM[+16.0]VEELNSGK[+8] | | 1015.477 | Oxidized M; Heavy K | | 2 |
| DBNL | VAGTGEGGLEEM[+16.0]VEELN[+1.0]SGK[+8] | | 1015.969 | Oxidized M; deamidated N; Heavy K | | 2 |
| DBNL | VAGTGEGGLEEM[+32.0]VEELNSGK[+8] | | 1023.475 | Dioxidized M; Heavy K | | 2 |
| DBNL | VAGTGEGGLEEM[+32.0]VEELN[+1.0]SGK | | 1023.967 | Dioxidized M; deamidated N; Heavy K | | 2 |
| DBNL | FQDVGPQAPVGSVYQK | 860.439 | | None | | 2 |
| DBNL | FQ[+1.0]DVGPQAPVGSVYQK | 860.931 | | Deamidated Q | | 2 |
| DBNL | FQ[+1.0]DVGPQ[+1.0]APVGSVYQK | 861.423 | | Deamidated Q | | 2 |
| DBNL | FQ[+1.0]DVGPQ[+1.0]APVGSVYQ[+1.0]K | 861.915 | | Deamidated Q | | 2 |
| TXNIP | SFEVVFNDPEK | 655.817 | | None | | 2 |
| TXNIP | SFEVVFN[+1.0]DPEK | 656.309 | | Deamidated N | | 2 |
| TXNIP | HTYLANGQTK | 566.791 | | None | | 2 |
| TXNIP | HTYLAN[+1.0]GQTK | 567.283 | | Deamidated N | | 2 |
| TXNIP | HTYLAN[+1.0]GQ[+1.0]TK | 567.775 | | Deamidated Q; deamidated N | | 2 |
| DNAJB4 | EALC[+57.0]GC[+57.0]SINVPTLDGR | 881.417 | | Carbamidomethylated C | | 2 |
| DNAJB4 | EALC[+57.0]GC[+57.0]SIN[+1.0]VPTLDGR | 881.909 | | Carbamidomethylated C; deamidated N | | 2 |
| DNAJB4 | EALC[+57.0]GC[+57.0]SINVPTLDGR[+10] | 886.421 | | Carbamidomethylated C; Heavy R | | 2 |
| DNAJB4 | EALC[+57.0]GC[+57.0]SIN[+1.0]VPTLDGR[+10] | 886.913 | | Carbamidomethylated C; deamidated N; Heavy R | | 2 |
| DNAJB4 | IIGYGLPFPK | 552.826 | | None | | 2 |
| ChickenOVA | ELINSWVESQTNGIIR | 929.987 | | None | | 2 |
| ChickenOVA | ELINSWVESQTNGIIR[+10] | 934.991 | | Heavy R | | 2 |
| ChickenOVA | GGLEPINFQTAADQAR | 844.424 | | None | | 2 |
| ChickenOVA | GGLEPINFQTAADQAR[+10] | 849.428 | | Heavy R | | 2 |
| Bovine  ALB | LVNELTEFAK | 582.319 | | None | | 2 |
| Bovine  ALB | LVNELTEFAK[+8] | 586.326 | | Heavy K | | 2 |
| Bovine  ALB | HLVDEPQNLIK | 653.362 | | None | | 2 |
| Bovine  ALB | HLVDEPQNLIK[+8] | 657.369 | | Heavy K | | 2 |
| Bovine  ALB | LGEYGFQNALIVR | 740.401 | | None | | 2 |
| Bovine  ALB | LGEYGFQNALIVR[+10] | 745.405 | | Heavy R | | 2 |

**Table S3.** Scheduled SRM parameters of light peptides monitored during method development.

| Compound | Start Time (min) | End Time (min) | Polarity | Precursor (m/z) | Product (m/z) | Collision Energy (V) | Dwell Time (ms) |
| --- | --- | --- | --- | --- | --- | --- | --- |
| sp\|O75608\|LYPA1  HUMAN  ASFPQGPIGGANR | 12 | 19.465 | Positive | 636.328 | 474.241 | 24.949 | 20 |
| sp\|O75608\|LYPA1  HUMAN  ASFPQGPIGGANR | 12 | 19.465 | Positive | 636.328 | 587.325 | 24.949 | 20 |
| sp\|O75608\|LYPA1_HUMAN_ASFPQGPIGGANR | 12 | 19.465 | Positive | 636.328 | 684.378 | 24.949 | 20 |
| sp\|O75608\|LYPA1_HUMAN_ASFPQGPIGGANR | 12 | 19.465 | Positive | 636.328 | 741.400 | 24.949 | 20 |
| sp\|O75608\|LYPA1_HUMAN_ASFPQGPIGGANR | 12 | 19.465 | Positive | 636.328 | 869.458 | 24.949 | 20 |
| sp\|O75608\|LYPA1_HUMAN_ASFPQGPIGGANR | 12 | 19.465 | Positive | 636.328 | 966.511 | 24.949 | 20 |
| sp\|O75608\|LYPA1_HUMAN_ASFPQGPIGGANR | 12 | 19.465 | Positive | 636.328 | 1113.579 | 24.949 | 20 |
| sp\|O75608\|LYPA1_HUMAN_LAGVTALSCWLPLR | 40.748 | 45.748 | Positive | 778.935 | 385.255 | 29.798 | 20 |
| sp\|O75608\|LYPA1_HUMAN_LAGVTALSCWLPLR | 40.748 | 45.748 | Positive | 778.935 | 684.419 | 29.798 | 20 |
| sp\|O75608\|LYPA1_HUMAN_LAGVTALSCWLPLR | 40.748 | 45.748 | Positive | 778.935 | 844.449 | 29.798 | 20 |
| sp\|O75608\|LYPA1_HUMAN_LAGVTALSCWLPLR | 40.748 | 45.748 | Positive | 778.935 | 931.481 | 29.798 | 20 |
| sp\|O75608\|LYPA1_HUMAN_LAGVTALSCWLPLR | 40.748 | 45.748 | Positive | 778.935 | 1044.565 | 29.798 | 20 |
| sp\|O75608\|LYPA1_HUMAN_LAGVTALSCWLPLR | 40.748 | 45.748 | Positive | 778.935 | 1115.602 | 29.798 | 20 |
| sp\|O75608\|LYPA1_HUMAN_LAGVTALSCWLPLR | 40.748 | 45.748 | Positive | 778.935 | 1216.650 | 29.798 | 20 |
| sp\|P00533\|EGFR_HUMAN_EISDGDVIISGNK | 14.425 | 19.425 | Positive | 673.844 | 518.293 | 26.225 | 20 |
| sp\|P00533\|EGFR_HUMAN_EISDGDVIISGNK | 14.425 | 19.425 | Positive | 673.844 | 631.377 | 26.225 | 20 |
| sp\|P00533\|EGFR_HUMAN_EISDGDVIISGNK | 14.425 | 19.425 | Positive | 673.844 | 730.445 | 26.225 | 20 |
| sp\|P00533\|EGFR_HUMAN_EISDGDVIISGNK | 14.425 | 19.425 | Positive | 673.844 | 845.472 | 26.225 | 20 |
| sp\|P00533\|EGFR_HUMAN_EISDGDVIISGNK | 14.425 | 19.425 | Positive | 673.844 | 902.494 | 26.225 | 20 |
| sp\|P00533\|EGFR_HUMAN_EISDGDVIISGNK | 14.425 | 19.425 | Positive | 673.844 | 1017.521 | 26.225 | 20 |
| sp\|P00533\|EGFR_HUMAN_EISDGDVIISGNK | 14.425 | 19.425 | Positive | 673.844 | 1104.553 | 26.225 | 20 |
| sp\|P00533\|EGFR_HUMAN_ELVEPLTPSGEAPNQALLR | 25 | 31.022 | Positive | 1017.547 | 600.382 | 37.911 | 20 |
| sp\|P00533\|EGFR_HUMAN_ELVEPLTPSGEAPNQALLR | 25 | 31.022 | Positive | 1017.547 | 714.425 | 37.911 | 20 |
| sp\|P00533\|EGFR_HUMAN_ELVEPLTPSGEAPNQALLR | 25 | 31.022 | Positive | 1017.547 | 811.478 | 37.911 | 20 |
| sp\|P00533\|EGFR_HUMAN_ELVEPLTPSGEAPNQALLR | 25 | 31.022 | Positive | 1017.547 | 882.515 | 37.911 | 20 |
| sp\|P00533\|EGFR_HUMAN_ELVEPLTPSGEAPNQALLR | 25 | 31.022 | Positive | 1017.547 | 1011.558 | 37.911 | 20 |
| sp\|P00533\|EGFR_HUMAN_ELVEPLTPSGEAPNQALLR | 25 | 31.022 | Positive | 1017.547 | 1068.579 | 37.911 | 20 |
| sp\|P00533\|EGFR_HUMAN_ELVEPLTPSGEAPNQALLR | 25 | 31.022 | Positive | 1017.547 | 1252.664 | 37.911 | 20 |
| sp\|P07996\|TSP1_HUMAN_GGVNDNFQGVLQNVR | 25.738 | 29.73817 | Positive | 808.911 | 629.372 | 30.817 | 20 |
| sp\|P07996\|TSP1_HUMAN_GGVNDNFQGVLQNVR | 25.738 | 29.73817 | Positive | 808.911 | 728.441 | 30.817 | 20 |
| sp\|P07996\|TSP1_HUMAN_GGVNDNFQGVLQNVR | 25.738 | 29.73817 | Positive | 808.911 | 785.462 | 30.817 | 20 |
| sp\|P07996\|TSP1_HUMAN_GGVNDNFQGVLQNVR | 25.738 | 29.73817 | Positive | 808.911 | 913.521 | 30.817 | 20 |
| sp\|P07996\|TSP1_HUMAN_GGVNDNFQGVLQNVR | 25.738 | 29.73817 | Positive | 808.911 | 1060.589 | 30.817 | 20 |
| sp\|P07996\|TSP1_HUMAN_GGVNDNFQGVLQNVR | 25.738 | 29.73817 | Positive | 808.911 | 1174.632 | 30.817 | 20 |
| sp\|P07996\|TSP1_HUMAN_GGVNDNFQGVLQNVR | 25.738 | 29.73817 | Positive | 808.911 | 1289.659 | 30.817 | 20 |
| sp\|P07996\|TSP1_HUMAN_TIVTTLQDSIR | 27.362 | 31.362 | Positive | 623.854 | 490.261 | 24.525 | 20 |
| sp\|P07996\|TSP1_HUMAN_TIVTTLQDSIR | 27.362 | 31.362 | Positive | 623.854 | 618.320 | 24.525 | 20 |
| sp\|P07996\|TSP1_HUMAN_TIVTTLQDSIR | 27.362 | 31.362 | Positive | 623.854 | 731.404 | 24.525 | 20 |
| sp\|P07996\|TSP1_HUMAN_TIVTTLQDSIR | 27.362 | 31.362 | Positive | 623.854 | 832.452 | 24.525 | 20 |
| sp\|P07996\|TSP1_HUMAN_TIVTTLQDSIR | 27.362 | 31.362 | Positive | 623.854 | 933.500 | 24.525 | 20 |
| sp\|P07996\|TSP1_HUMAN_TIVTTLQDSIR | 27.362 | 31.362 | Positive | 623.854 | 1032.568 | 24.525 | 20 |
| sp\|P07996\|TSP1_HUMAN_TIVTTLQDSIR | 27.362 | 31.362 | Positive | 623.854 | 1145.652 | 24.525 | 20 |
| sp\|P09486\|SPRC_HUMAN_FFETCDLDNDK | 17 | 23.054 | Positive | 702.293 | 491.209 | 27.192 | 20 |
| sp\|P09486\|SPRC_HUMAN_FFETCDLDNDK | 17 | 23.054 | Positive | 702.293 | 604.293 | 27.192 | 20 |
| sp\|P09486\|SPRC_HUMAN_FFETCDLDNDK | 17 | 23.054 | Positive | 702.293 | 719.320 | 27.192 | 20 |
| sp\|P09486\|SPRC_HUMAN_FFETCDLDNDK | 17 | 23.054 | Positive | 702.293 | 879.351 | 27.192 | 20 |
| sp\|P09486\|SPRC_HUMAN_FFETCDLDNDK | 17 | 23.054 | Positive | 702.293 | 980.398 | 27.192 | 20 |
| sp\|P09486\|SPRC_HUMAN_FFETCDLDNDK | 17 | 23.054 | Positive | 702.293 | 1109.441 | 27.192 | 20 |
| sp\|P09486\|SPRC_HUMAN_FFETCDLDNDK | 17 | 23.054 | Positive | 702.293 | 1256.509 | 27.192 | 20 |
| sp\|P09486\|SPRC_HUMAN_YIPPCLDSELTEFPLR | 35.657 | 40.657 | Positive | 975.487 | 385.255 | 36.481 | 20 |
| sp\|P09486\|SPRC_HUMAN_YIPPCLDSELTEFPLR | 35.657 | 40.657 | Positive | 975.487 | 532.324 | 36.481 | 20 |
| sp\|P09486\|SPRC_HUMAN_YIPPCLDSELTEFPLR | 35.657 | 40.657 | Positive | 975.487 | 661.366 | 36.481 | 20 |
| sp\|P09486\|SPRC_HUMAN_YIPPCLDSELTEFPLR | 35.657 | 40.657 | Positive | 975.487 | 762.414 | 36.481 | 20 |
| sp\|P09486\|SPRC_HUMAN_YIPPCLDSELTEFPLR | 35.657 | 40.657 | Positive | 975.487 | 875.498 | 36.481 | 20 |
| sp\|P09486\|SPRC_HUMAN_YIPPCLDSELTEFPLR | 35.657 | 40.657 | Positive | 975.487 | 1004.541 | 36.481 | 20 |
| sp\|P09486\|SPRC_HUMAN_YIPPCLDSELTEFPLR | 35.657 | 40.657 | Positive | 975.487 | 1091.573 | 36.481 | 20 |
| sp\|P09601\|HMOX1_HUMAN_TEPELLVAHAYTR | 20.822 | 24.822 | Positive | 750.396 | 647.325 | 28.827 | 20 |
| sp\|P09601\|HMOX1_HUMAN_TEPELLVAHAYTR | 20.822 | 24.822 | Positive | 750.396 | 718.363 | 28.827 | 20 |
| sp\|P09601\|HMOX1_HUMAN_TEPELLVAHAYTR | 20.822 | 24.822 | Positive | 750.396 | 817.431 | 28.827 | 20 |
| sp\|P09601\|HMOX1_HUMAN_TEPELLVAHAYTR | 20.822 | 24.822 | Positive | 750.396 | 930.515 | 28.827 | 20 |
| sp\|P09601\|HMOX1_HUMAN_TEPELLVAHAYTR | 20.822 | 24.822 | Positive | 750.396 | 1043.599 | 28.827 | 20 |
| sp\|P09601\|HMOX1_HUMAN_TEPELLVAHAYTR | 20.822 | 24.822 | Positive | 750.396 | 1172.642 | 28.827 | 20 |
| sp\|P09601\|HMOX1_HUMAN_TEPELLVAHAYTR | 20.822 | 24.822 | Positive | 750.396 | 1269.694 | 28.827 | 20 |
| sp\|P09601\|HMOX1_HUMAN_VQDSAPVETPR | 0 | 12.078 | Positive | 599.807 | 601.330 | 23.707 | 20 |
| sp\|P09601\|HMOX1_HUMAN_VQDSAPVETPR | 0 | 12.078 | Positive | 599.807 | 698.383 | 23.707 | 20 |
| sp\|P09601\|HMOX1_HUMAN_VQDSAPVETPR | 0 | 12.078 | Positive | 599.807 | 769.420 | 23.707 | 20 |
| sp\|P09601\|HMOX1_HUMAN_VQDSAPVETPR | 0 | 12.078 | Positive | 599.807 | 856.452 | 23.707 | 20 |
| sp\|P09601\|HMOX1_HUMAN_VQDSAPVETPR | 0 | 12.078 | Positive | 599.807 | 971.479 | 23.707 | 20 |
| sp\|P09601\|HMOX1_HUMAN_VQDSAPVETPR | 0 | 12.078 | Positive | 599.807 | 1099.537 | 23.707 | 20 |
| sp\|P15104\|GLNA_HUMAN_LTGFHETSNINDFSAGVANR | 0 | 60 | Positive | 1075.517 | 1277.623 | 35.2 | 20 |
| sp\|P15104\|GLNA_HUMAN_LTGFHETSNINDFSAGVANR | 0 | 60 | Positive | 1075.517 | 1163.580 | 35.2 | 20 |
| sp\|P15104\|GLNA_HUMAN_LTGFHETSNINDFSAGVANR | 0 | 60 | Positive | 1075.517 | 1050.496 | 35.2 | 20 |
| sp\|P15104\|GLNA_HUMAN_LTGFHETSNINDFSAGVANR | 0 | 60 | Positive | 1075.517 | 936.453 | 35.2 | 20 |
| sp\|P15104\|GLNA_HUMAN_LTGFHETSNINDFSAGVANR | 0 | 60 | Positive | 1075.517 | 821.426 | 35.2 | 20 |
| sp\|P15104\|GLNA_HUMAN_LTGFHETSNINDFSAGVANR | 0 | 60 | Positive | 1075.517 | 674.358 | 35.2 | 20 |
| sp\|P15104\|GLNA_HUMAN_LTGFHETSNINDFSAGVANR | 0 | 60 | Positive | 1075.517 | 587.326 | 35.2 | 20 |
| sp\|P15104\|GLNA_HUMAN_LTGFHETSNINDFSAGVANR | 0 | 60 | Positive | 1075.517 | 516.289 | 35.2 | 20 |
| sp\|P15104\|GLNA_HUMAN_LVLCEVFK | 27 | 32.835 | Positive | 504.283 | 393.249 | 20.46 | 20 |
| sp\|P15104\|GLNA_HUMAN_LVLCEVFK | 27 | 32.835 | Positive | 504.283 | 522.292 | 20.46 | 20 |
| sp\|P15104\|GLNA_HUMAN_LVLCEVFK | 27 | 32.835 | Positive | 504.283 | 682.322 | 20.46 | 20 |
| sp\|P15104\|GLNA_HUMAN_LVLCEVFK | 27 | 32.835 | Positive | 504.283 | 795.406 | 20.46 | 20 |
| sp\|P15104\|GLNA_HUMAN_LVLCEVFK | 27 | 32.835 | Positive | 504.283 | 894.475 | 20.46 | 20 |
| sp\|P15104\|GLNA_HUMAN_TC[+57.0]LLNETGDEPFQYK | 0 | 60 | Positive | 907.917 | 1213.537 | 30.1 | 20 |
| sp\|P15104\|GLNA_HUMAN_TC[+57.0]LLNETGDEPFQYK | 0 | 60 | Positive | 907.917 | 1084.495 | 30.1 | 20 |
| sp\|P15104\|GLNA_HUMAN_TC[+57.0]LLNETGDEPFQYK | 0 | 60 | Positive | 907.917 | 983.447 | 30.1 | 20 |
| sp\|P15104\|GLNA_HUMAN_TC[+57.0]LLNETGDEPFQYK | 0 | 60 | Positive | 907.917 | 926.425 | 30.1 | 20 |
| sp\|P15104\|GLNA_HUMAN_TC[+57.0]LLNETGDEPFQYK | 0 | 60 | Positive | 907.917 | 811.398 | 30.1 | 20 |
| sp\|P15104\|GLNA_HUMAN_TC[+57.0]LLNETGDEPFQYK | 0 | 60 | Positive | 907.917 | 682.356 | 30.1 | 20 |
| sp\|P15104\|GLNA_HUMAN_TC[+57.0]LLNETGDEPFQYK | 0 | 60 | Positive | 907.917 | 585.303 | 30.1 | 20 |
| sp\|P37173\|TGFR2_HUMAN_IFPYEEYASWK | 28.819 | 33.819 | Positive | 716.843 | 420.224 | 27.687 | 20 |
| sp\|P37173\|TGFR2_HUMAN_IFPYEEYASWK | 28.819 | 33.819 | Positive | 716.843 | 491.261 | 27.687 | 20 |
| sp\|P37173\|TGFR2_HUMAN_IFPYEEYASWK | 28.819 | 33.819 | Positive | 716.843 | 654.324 | 27.687 | 20 |
| sp\|P37173\|TGFR2_HUMAN_IFPYEEYASWK | 28.819 | 33.819 | Positive | 716.843 | 783.367 | 27.687 | 20 |
| sp\|P37173\|TGFR2_HUMAN_IFPYEEYASWK | 28.819 | 33.819 | Positive | 716.843 | 912.409 | 27.687 | 20 |
| sp\|P37173\|TGFR2_HUMAN_IFPYEEYASWK | 28.819 | 33.819 | Positive | 716.843 | 1075.473 | 27.687 | 20 |
| sp\|P37173\|TGFR2_HUMAN_IFPYEEYASWK | 28.819 | 33.819 | Positive | 716.843 | 1172.525 | 27.687 | 20 |
| sp\|P37173\|TGFR2_HUMAN_LDPTLSVDDLANSGQVGTAR | 24.73 | 29.73 | Positive | 1015.013 | 503.293 | 37.824 | 20 |
| sp\|P37173\|TGFR2_HUMAN_LDPTLSVDDLANSGQVGTAR | 24.73 | 29.73 | Positive | 1015.013 | 631.352 | 37.824 | 20 |
| sp\|P37173\|TGFR2_HUMAN_LDPTLSVDDLANSGQVGTAR | 24.73 | 29.73 | Positive | 1015.013 | 688.373 | 37.824 | 20 |
| sp\|P37173\|TGFR2_HUMAN_LDPTLSVDDLANSGQVGTAR | 24.73 | 29.73 | Positive | 1015.013 | 775.405 | 37.824 | 20 |
| sp\|P37173\|TGFR2_HUMAN_LDPTLSVDDLANSGQVGTAR | 24.73 | 29.73 | Positive | 1015.013 | 889.448 | 37.824 | 20 |
| sp\|P37173\|TGFR2_HUMAN_LDPTLSVDDLANSGQVGTAR | 24.73 | 29.73 | Positive | 1015.013 | 960.485 | 37.824 | 20 |
| sp\|P37173\|TGFR2_HUMAN_LDPTLSVDDLANSGQVGTAR | 24.73 | 29.73 | Positive | 1015.013 | 1073.569 | 37.824 | 20 |
| sp\|P55268\|LAMB2_HUMAN_GSCYPATGDLLVGR | 21.184 | 26.184 | Positive | 733.359 | 444.292 | 28.248 | 20 |
| sp\|P55268\|LAMB2_HUMAN_GSCYPATGDLLVGR | 21.184 | 26.184 | Positive | 733.359 | 557.376 | 28.248 | 20 |
| sp\|P55268\|LAMB2_HUMAN_GSCYPATGDLLVGR | 21.184 | 26.184 | Positive | 733.359 | 672.403 | 28.248 | 20 |
| sp\|P55268\|LAMB2_HUMAN_GSCYPATGDLLVGR | 21.184 | 26.184 | Positive | 733.359 | 729.425 | 28.248 | 20 |
| sp\|P55268\|LAMB2_HUMAN_GSCYPATGDLLVGR | 21.184 | 26.184 | Positive | 733.359 | 830.473 | 28.248 | 20 |
| sp\|P55268\|LAMB2_HUMAN_GSCYPATGDLLVGR | 21.184 | 26.184 | Positive | 733.359 | 901.510 | 28.248 | 20 |
| sp\|P55268\|LAMB2_HUMAN_GSCYPATGDLLVGR | 21.184 | 26.184 | Positive | 733.359 | 998.562 | 28.248 | 20 |
| sp\|P55268\|LAMB2_HUMAN_GSCYPATGDLLVGR | 21.184 | 26.184 | Positive | 733.359 | 1161.626 | 28.248 | 20 |
| sp\|P55268\|LAMB2_HUMAN_LQEGQTLEFLVASVPK | 37.298 | 42.298 | Positive | 879.985 | 501.303 | 33.234 | 20 |
| sp\|P55268\|LAMB2_HUMAN_LQEGQTLEFLVASVPK | 37.298 | 42.298 | Positive | 879.985 | 600.371 | 33.234 | 20 |
| sp\|P55268\|LAMB2_HUMAN_LQEGQTLEFLVASVPK | 37.298 | 42.298 | Positive | 879.985 | 713.455 | 33.234 | 20 |
| sp\|P55268\|LAMB2_HUMAN_LQEGQTLEFLVASVPK | 37.298 | 42.298 | Positive | 879.985 | 860.523 | 33.234 | 20 |
| sp\|P55268\|LAMB2_HUMAN_LQEGQTLEFLVASVPK | 37.298 | 42.298 | Positive | 879.985 | 989.566 | 33.234 | 20 |
| sp\|P55268\|LAMB2_HUMAN_LQEGQTLEFLVASVPK | 37.298 | 42.298 | Positive | 879.985 | 1102.650 | 33.234 | 20 |
| sp\|P55268\|LAMB2_HUMAN_LQEGQTLEFLVASVPK | 37.298 | 42.298 | Positive | 879.985 | 1203.698 | 33.234 | 20 |
| sp\|P62745\|RHOB_HUMAN_EVFETATR | 0 | 12 | Positive | 476.740 | 823.431 | 17.2 | 20 |
| sp\|P62745\|RHOB_HUMAN_EVFETATR | 0 | 12 | Positive | 476.740 | 724.362 | 17.2 | 20 |
| sp\|P62745\|RHOB_HUMAN_EVFETATR | 0 | 12 | Positive | 476.740 | 577.294 | 17.2 | 20 |
| sp\|P62745\|RHOB_HUMAN_EVFETATR | 0 | 12 | Positive | 476.740 | 448.251 | 17.2 | 20 |
| sp\|P62745\|RHOB_HUMAN_EVFETATR | 0 | 12 | Positive | 476.740 | 347.204 | 17.2 | 20 |
| sp\|P62745\|RHOB_HUMAN_IQAYDYLEC[+57.0]SAK | 10 | 30 | Positive | 730.840 | 1219.530 | 24.8 | 20 |
| sp\|P62745\|RHOB_HUMAN_IQAYDYLEC[+57.0]SAK | 10 | 30 | Positive | 730.840 | 1148.493 | 24.8 | 20 |
| sp\|P62745\|RHOB_HUMAN_IQAYDYLEC[+57.0]SAK | 10 | 30 | Positive | 730.840 | 985.430 | 24.8 | 20 |
| sp\|P62745\|RHOB_HUMAN_IQAYDYLEC[+57.0]SAK | 10 | 30 | Positive | 730.840 | 870.403 | 24.8 | 20 |
| sp\|P62745\|RHOB_HUMAN_IQAYDYLEC[+57.0]SAK | 10 | 30 | Positive | 730.840 | 707.339 | 24.8 | 20 |
| sp\|P62745\|RHOB_HUMAN_IQAYDYLEC[+57.0]SAK | 10 | 30 | Positive | 730.840 | 594.255 | 24.8 | 20 |
| sp\|P62745\|RHOB_HUMAN_IQAYDYLEC[+57.0]SAK | 10 | 30 | Positive | 730.840 | 465.213 | 24.8 | 20 |
| sp\|P62745\|RHOB_HUMAN_LVVVGDGACGK | 10 | 15.66718 | Positive | 537.784 | 435.202 | 21.599 | 20 |
| sp\|P62745\|RHOB_HUMAN_LVVVGDGACGK | 10 | 15.66718 | Positive | 537.784 | 492.223 | 21.599 | 20 |
| sp\|P62745\|RHOB_HUMAN_LVVVGDGACGK | 10 | 15.66718 | Positive | 537.784 | 607.250 | 21.599 | 20 |
| sp\|P62745\|RHOB_HUMAN_LVVVGDGACGK | 10 | 15.66718 | Positive | 537.784 | 664.271 | 21.599 | 20 |
| sp\|P62745\|RHOB_HUMAN_LVVVGDGACGK | 10 | 15.66718 | Positive | 537.784 | 763.340 | 21.599 | 20 |
| sp\|P62745\|RHOB_HUMAN_LVVVGDGACGK | 10 | 15.66718 | Positive | 537.784 | 862.408 | 21.599 | 20 |
| sp\|P62745\|RHOB_HUMAN_LVVVGDGACGK | 10 | 15.66718 | Positive | 537.784 | 961.477 | 21.599 | 20 |
| sp\|P98179\|RBM3_HUMAN_GGGDQGYGSGR | 0 | 7.095 | Positive | 505.718 | 376.193 | 20.508 | 20 |
| sp\|P98179\|RBM3_HUMAN_GGGDQGYGSGR | 0 | 7.095 | Positive | 505.718 | 539.257 | 20.508 | 20 |
| sp\|P98179\|RBM3_HUMAN_GGGDQGYGSGR | 0 | 7.095 | Positive | 505.718 | 596.278 | 20.508 | 20 |
| sp\|P98179\|RBM3_HUMAN_GGGDQGYGSGR | 0 | 7.095 | Positive | 505.718 | 724.337 | 20.508 | 20 |
| sp\|P98179\|RBM3_HUMAN_GGGDQGYGSGR | 0 | 7.095 | Positive | 505.718 | 839.364 | 20.508 | 20 |
| sp\|P98179\|RBM3_HUMAN_GGGDQGYGSGR | 0 | 7.095 | Positive | 505.718 | 896.385 | 20.508 | 20 |
| sp\|P98179\|RBM3_HUMAN_GGGDQGYGSGR | 0 | 7.095 | Positive | 505.718 | 953.407 | 20.508 | 20 |
| sp\|P98179\|RBM3_HUMAN_YYDSRPGGYGYGYGR | 11.511 | 16.511 | Positive | 865.881 | 615.288 | 32.754 | 20 |
| sp\|P98179\|RBM3_HUMAN_YYDSRPGGYGYGYGR | 11.511 | 16.511 | Positive | 865.881 | 672.309 | 32.754 | 20 |
| sp\|P98179\|RBM3_HUMAN_YYDSRPGGYGYGYGR | 11.511 | 16.511 | Positive | 865.881 | 835.373 | 32.754 | 20 |
| sp\|P98179\|RBM3_HUMAN_YYDSRPGGYGYGYGR | 11.511 | 16.511 | Positive | 865.881 | 892.394 | 32.754 | 20 |
| sp\|P98179\|RBM3_HUMAN_YYDSRPGGYGYGYGR | 11.511 | 16.511 | Positive | 865.881 | 949.416 | 32.754 | 20 |
| sp\|P98179\|RBM3_HUMAN_YYDSRPGGYGYGYGR | 11.511 | 16.511 | Positive | 865.881 | 1046.469 | 32.754 | 20 |
| sp\|P98179\|RBM3_HUMAN_YYDSRPGGYGYGYGR | 11.511 | 16.511 | Positive | 865.881 | 1202.570 | 32.754 | 20 |
| sp\|Q10588\|BST1_HUMAN_AGLIIPLFLVLASR | 51.785 | 56.785 | Positive | 741.974 | 446.272 | 28.541 | 20 |
| sp\|Q10588\|BST1_HUMAN_AGLIIPLFLVLASR | 51.785 | 56.785 | Positive | 741.974 | 545.340 | 28.541 | 20 |
| sp\|Q10588\|BST1_HUMAN_AGLIIPLFLVLASR | 51.785 | 56.785 | Positive | 741.974 | 658.424 | 28.541 | 20 |
| sp\|Q10588\|BST1_HUMAN_AGLIIPLFLVLASR | 51.785 | 56.785 | Positive | 741.974 | 805.493 | 28.541 | 20 |
| sp\|Q10588\|BST1_HUMAN_AGLIIPLFLVLASR | 51.785 | 56.785 | Positive | 741.974 | 918.577 | 28.541 | 20 |
| sp\|Q10588\|BST1_HUMAN_AGLIIPLFLVLASR | 51.785 | 56.785 | Positive | 741.974 | 1015.629 | 28.541 | 20 |
| sp\|Q10588\|BST1_HUMAN_AGLIIPLFLVLASR | 51.785 | 56.785 | Positive | 741.974 | 1128.713 | 28.541 | 20 |
| sp\|Q10588\|BST1_HUMAN_AGLIIPLFLVLASR | 51.785 | 56.785 | Positive | 741.974 | 1241.797 | 28.541 | 20 |
| sp\|Q10588\|BST1_HUMAN_GFFADYEIPNLQK | 31 | 37.016 | Positive | 771.385 | 502.298 | 29.541 | 20 |
| sp\|Q10588\|BST1_HUMAN_GFFADYEIPNLQK | 31 | 37.016 | Positive | 771.385 | 599.351 | 29.541 | 20 |
| sp\|Q10588\|BST1_HUMAN_GFFADYEIPNLQK | 31 | 37.016 | Positive | 771.385 | 712.435 | 29.541 | 20 |
| sp\|Q10588\|BST1_HUMAN_GFFADYEIPNLQK | 31 | 37.016 | Positive | 771.385 | 841.477 | 29.541 | 20 |
| sp\|Q10588\|BST1_HUMAN_GFFADYEIPNLQK | 31 | 37.016 | Positive | 771.385 | 1004.541 | 29.541 | 20 |
| sp\|Q10588\|BST1_HUMAN_GFFADYEIPNLQK | 31 | 37.016 | Positive | 771.385 | 1119.568 | 29.541 | 20 |
| sp\|Q10588\|BST1_HUMAN_GFFADYEIPNLQK | 31 | 37.016 | Positive | 771.385 | 1190.605 | 29.541 | 20 |
| sp\|Q53EL6\|PDCD4_HUMAN_APQLVGQFIAR | 25.267 | 29.267 | Positive | 600.348 | 506.308 | 23.726 | 20 |
| sp\|Q53EL6\|PDCD4_HUMAN_APQLVGQFIAR | 25.267 | 29.267 | Positive | 600.348 | 634.367 | 23.726 | 20 |
| sp\|Q53EL6\|PDCD4_HUMAN_APQLVGQFIAR | 25.267 | 29.267 | Positive | 600.348 | 691.388 | 23.726 | 20 |
| sp\|Q53EL6\|PDCD4_HUMAN_APQLVGQFIAR | 25.267 | 29.267 | Positive | 600.348 | 790.456 | 23.726 | 20 |
| sp\|Q53EL6\|PDCD4_HUMAN_APQLVGQFIAR | 25.267 | 29.267 | Positive | 600.348 | 903.541 | 23.726 | 20 |
| sp\|Q53EL6\|PDCD4_HUMAN_APQLVGQFIAR | 25.267 | 29.267 | Positive | 600.348 | 1031.599 | 23.726 | 20 |
| sp\|Q53EL6\|PDCD4_HUMAN_APQLVGQFIAR | 25.267 | 29.267 | Positive | 600.348 | 1128.652 | 23.726 | 20 |
| sp\|Q53EL6\|PDCD4_HUMAN_SGVPVLAVSLALEGK | 32 | 38.59878 | Positive | 720.427 | 630.382 | 27.809 | 20 |
| sp\|Q53EL6\|PDCD4_HUMAN_SGVPVLAVSLALEGK | 32 | 38.59878 | Positive | 720.427 | 717.414 | 27.809 | 20 |
| sp\|Q53EL6\|PDCD4_HUMAN_SGVPVLAVSLALEGK | 32 | 38.59878 | Positive | 720.427 | 816.482 | 27.809 | 20 |
| sp\|Q53EL6\|PDCD4_HUMAN_SGVPVLAVSLALEGK | 32 | 38.59878 | Positive | 720.427 | 887.519 | 27.809 | 20 |
| sp\|Q53EL6\|PDCD4_HUMAN_SGVPVLAVSLALEGK | 32 | 38.59878 | Positive | 720.427 | 1000.603 | 27.809 | 20 |
| sp\|Q53EL6\|PDCD4_HUMAN_SGVPVLAVSLALEGK | 32 | 38.59878 | Positive | 720.427 | 1099.672 | 27.809 | 20 |
| sp\|Q53EL6\|PDCD4_HUMAN_SGVPVLAVSLALEGK | 32 | 38.59878 | Positive | 720.427 | 1196.724 | 27.809 | 20 |
| sp\|Q8WV24\|PHLA1_HUMAN_AAGNGEAEPSGGPSYAGR | 0 | 10.533 | Positive | 824.372 | 650.325 | 31.343 | 20 |
| sp\|Q8WV24\|PHLA1_HUMAN_AAGNGEAEPSGGPSYAGR | 0 | 10.533 | Positive | 824.372 | 707.347 | 31.343 | 20 |
| sp\|Q8WV24\|PHLA1_HUMAN_AAGNGEAEPSGGPSYAGR | 0 | 10.533 | Positive | 824.372 | 764.368 | 31.343 | 20 |
| sp\|Q8WV24\|PHLA1_HUMAN_AAGNGEAEPSGGPSYAGR | 0 | 10.533 | Positive | 824.372 | 851.400 | 31.343 | 20 |
| sp\|Q8WV24\|PHLA1_HUMAN_AAGNGEAEPSGGPSYAGR | 0 | 10.533 | Positive | 824.372 | 948.453 | 31.343 | 20 |
| sp\|Q8WV24\|PHLA1_HUMAN_AAGNGEAEPSGGPSYAGR | 0 | 10.533 | Positive | 824.372 | 1077.495 | 31.343 | 20 |
| sp\|Q8WV24\|PHLA1_HUMAN_AAGNGEAEPSGGPSYAGR | 0 | 10.533 | Positive | 824.372 | 1148.532 | 31.343 | 20 |
| sp\|Q8WV24\|PHLA1_HUMAN_AAGNGEAEPSGGPSYAGR | 0 | 10.533 | Positive | 824.372 | 1277.575 | 31.343 | 20 |
| sp\|Q92974\|ARHG2_HUMAN_DLLVGPGVELLLTPR | 40 | 46.523 | Positive | 796.474 | 599.387 | 30.394 | 20 |
| sp\|Q92974\|ARHG2_HUMAN_DLLVGPGVELLLTPR | 40 | 46.523 | Positive | 796.474 | 712.471 | 30.394 | 20 |
| sp\|Q92974\|ARHG2_HUMAN_DLLVGPGVELLLTPR | 40 | 46.523 | Positive | 796.474 | 841.514 | 30.394 | 20 |
| sp\|Q92974\|ARHG2_HUMAN_DLLVGPGVELLLTPR | 40 | 46.523 | Positive | 796.474 | 940.582 | 30.394 | 20 |
| sp\|Q92974\|ARHG2_HUMAN_DLLVGPGVELLLTPR | 40 | 46.523 | Positive | 796.474 | 997.604 | 30.394 | 20 |
| sp\|Q92974\|ARHG2_HUMAN_DLLVGPGVELLLTPR | 40 | 46.523 | Positive | 796.474 | 1094.656 | 30.394 | 20 |
| sp\|Q92974\|ARHG2_HUMAN_DLLVGPGVELLLTPR | 40 | 46.523 | Positive | 796.474 | 1151.678 | 30.394 | 20 |
| sp\|Q92974\|ARHG2_HUMAN_DLLVGPGVELLLTPR | 40 | 46.523 | Positive | 796.474 | 1250.746 | 30.394 | 20 |
| sp\|Q92974\|ARHG2_HUMAN_ELLSNVDEGIYQLEK | 24 | 40 | Positive | 875.449 | 1307.648 | 29.2 | 20 |
| sp\|Q92974\|ARHG2_HUMAN_ELLSNVDEGIYQLEK | 24 | 40 | Positive | 875.449 | 1193.605 | 29.2 | 20 |
| sp\|Q92974\|ARHG2_HUMAN_ELLSNVDEGIYQLEK | 24 | 40 | Positive | 875.449 | 1094.536 | 29.2 | 20 |
| sp\|Q92974\|ARHG2_HUMAN_ELLSNVDEGIYQLEK | 24 | 40 | Positive | 875.449 | 979.509 | 29.2 | 20 |
| sp\|Q92974\|ARHG2_HUMAN_ELLSNVDEGIYQLEK | 24 | 40 | Positive | 875.449 | 850.467 | 29.2 | 20 |
| sp\|Q92974\|ARHG2_HUMAN_ELLSNVDEGIYQLEK | 24 | 40 | Positive | 875.449 | 793.445 | 29.2 | 20 |
| sp\|Q92974\|ARHG2_HUMAN_ELLSNVDEGIYQLEK | 24 | 40 | Positive | 875.449 | 680.361 | 29.2 | 20 |
| sp\|Q92974\|ARHG2_HUMAN_ELLSNVDEGIYQLEK | 24 | 40 | Positive | 875.449 | 517.298 | 29.2 | 20 |
| sp\|Q96JH7\|VCIP1_HUMAN_SSGDYSATFLPGLIPAEK | 24 | 40 | Positive | 926.970 | 1084.640 | 30.7 | 20 |
| sp\|Q96JH7\|VCIP1_HUMAN_SSGDYSATFLPGLIPAEK | 24 | 40 | Positive | 926.970 | 937.572 | 30.7 | 20 |
| sp\|Q96JH7\|VCIP1_HUMAN_SSGDYSATFLPGLIPAEK | 24 | 40 | Positive | 926.970 | 824.488 | 30.7 | 20 |
| sp\|Q96JH7\|VCIP1_HUMAN_SSGDYSATFLPGLIPAEK | 24 | 40 | Positive | 926.970 | 727.435 | 30.7 | 20 |
| sp\|Q96JH7\|VCIP1_HUMAN_SSGDYSATFLPGLIPAEK | 24 | 40 | Positive | 926.970 | 670.413 | 30.7 | 20 |
| sp\|Q96JH7\|VCIP1_HUMAN_SSGDYSATFLPGLIPAEK | 24 | 40 | Positive | 926.970 | 557.329 | 30.7 | 20 |
| sp\|Q96JH7\|VCIP1_HUMAN_SSGDYSATFLPGLIPAEK | 24 | 40 | Positive | 926.970 | 444.245 | 30.7 | 20 |
| sp\|Q96JH7\|VCIP1_HUMAN_TEPSVFTASSSNSELIR | 22 | 28.168 | Positive | 912.952 | 617.361 | 34.354 | 20 |
| sp\|Q96JH7\|VCIP1_HUMAN_TEPSVFTASSSNSELIR | 22 | 28.168 | Positive | 912.952 | 731.404 | 34.354 | 20 |
| sp\|Q96JH7\|VCIP1_HUMAN_TEPSVFTASSSNSELIR | 22 | 28.168 | Positive | 912.952 | 818.436 | 34.354 | 20 |
| sp\|Q96JH7\|VCIP1_HUMAN_TEPSVFTASSSNSELIR | 22 | 28.168 | Positive | 912.952 | 905.468 | 34.354 | 20 |
| sp\|Q96JH7\|VCIP1_HUMAN_TEPSVFTASSSNSELIR | 22 | 28.168 | Positive | 912.952 | 992.500 | 34.354 | 20 |
| sp\|Q96JH7\|VCIP1_HUMAN_TEPSVFTASSSNSELIR | 22 | 28.168 | Positive | 912.952 | 1063.537 | 34.354 | 20 |
| sp\|Q96JH7\|VCIP1_HUMAN_TEPSVFTASSSNSELIR | 22 | 28.168 | Positive | 912.952 | 1164.585 | 34.354 | 20 |
| sp\|Q9H3M7\|TXNIP_HUMAN_HTYLANGQTK | 0 | 12 | Positive | 566.791 | 995.516 | 19.9 | 20 |
| sp\|Q9H3M7\|TXNIP_HUMAN_HTYLANGQTK | 0 | 12 | Positive | 566.791 | 894.468 | 19.9 | 20 |
| sp\|Q9H3M7\|TXNIP_HUMAN_HTYLANGQTK | 0 | 12 | Positive | 566.791 | 731.405 | 19.9 | 20 |
| sp\|Q9H3M7\|TXNIP_HUMAN_HTYLANGQTK | 0 | 12 | Positive | 566.791 | 618.321 | 19.9 | 20 |
| sp\|Q9H3M7\|TXNIP_HUMAN_HTYLANGQTK | 0 | 12 | Positive | 566.791 | 547.283 | 19.9 | 20 |
| sp\|Q9H3M7\|TXNIP_HUMAN_HTYLANGQTK | 0 | 12 | Positive | 566.791 | 433.241 | 19.9 | 20 |
| sp\|Q9H3M7\|TXNIP_HUMAN_HTYLANGQTK | 0 | 12 | Positive | 566.791 | 376.219 | 19.9 | 20 |
| sp\|Q9H3M7\|TXNIP_HUMAN_SFEVVFNDPEK | 21 | 28.756 | Positive | 655.817 | 373.208 | 25.612 | 20 |
| sp\|Q9H3M7\|TXNIP_HUMAN_SFEVVFNDPEK | 21 | 28.756 | Positive | 655.817 | 488.235 | 25.612 | 20 |
| sp\|Q9H3M7\|TXNIP_HUMAN_SFEVVFNDPEK | 21 | 28.756 | Positive | 655.817 | 602.278 | 25.612 | 20 |
| sp\|Q9H3M7\|TXNIP_HUMAN_SFEVVFNDPEK | 21 | 28.756 | Positive | 655.817 | 749.346 | 25.612 | 20 |
| sp\|Q9H3M7\|TXNIP_HUMAN_SFEVVFNDPEK | 21 | 28.756 | Positive | 655.817 | 848.414 | 25.612 | 20 |
| sp\|Q9H3M7\|TXNIP_HUMAN_SFEVVFNDPEK | 21 | 28.756 | Positive | 655.817 | 947.483 | 25.612 | 20 |
| sp\|Q9H3M7\|TXNIP_HUMAN_SFEVVFNDPEK | 21 | 28.756 | Positive | 655.817 | 1076.525 | 25.612 | 20 |
| sp\|Q9UDY4\|DNJB4_HUMAN_EALCGCSINVPTLDGR | 23 | 29.01 | Positive | 881.417 | 561.299 | 33.282 | 20 |
| sp\|Q9UDY4\|DNJB4_HUMAN_EALCGCSINVPTLDGR | 23 | 29.01 | Positive | 881.417 | 658.351 | 33.282 | 20 |
| sp\|Q9UDY4\|DNJB4_HUMAN_EALCGCSINVPTLDGR | 23 | 29.01 | Positive | 881.417 | 757.420 | 33.282 | 20 |
| sp\|Q9UDY4\|DNJB4_HUMAN_EALCGCSINVPTLDGR | 23 | 29.01 | Positive | 881.417 | 871.463 | 33.282 | 20 |
| sp\|Q9UDY4\|DNJB4_HUMAN_EALCGCSINVPTLDGR | 23 | 29.01 | Positive | 881.417 | 984.547 | 33.282 | 20 |
| sp\|Q9UDY4\|DNJB4_HUMAN_EALCGCSINVPTLDGR | 23 | 29.01 | Positive | 881.417 | 1071.579 | 33.282 | 20 |
| sp\|Q9UDY4\|DNJB4_HUMAN_EALCGCSINVPTLDGR | 23 | 29.01 | Positive | 881.417 | 1231.609 | 33.282 | 20 |
| sp\|Q9UDY4\|DNJB4_HUMAN_IIGYGLPFPK | 29 | 35.371 | Positive | 552.826 | 391.233 | 22.11 | 20 |
| sp\|Q9UDY4\|DNJB4_HUMAN_IIGYGLPFPK | 29 | 35.371 | Positive | 552.826 | 488.286 | 22.11 | 20 |
| sp\|Q9UDY4\|DNJB4_HUMAN_IIGYGLPFPK | 29 | 35.371 | Positive | 552.826 | 601.370 | 22.11 | 20 |
| sp\|Q9UDY4\|DNJB4_HUMAN_IIGYGLPFPK | 29 | 35.371 | Positive | 552.826 | 658.392 | 22.11 | 20 |
| sp\|Q9UDY4\|DNJB4_HUMAN_IIGYGLPFPK | 29 | 35.371 | Positive | 552.826 | 821.455 | 22.11 | 20 |
| sp\|Q9UDY4\|DNJB4_HUMAN_IIGYGLPFPK | 29 | 35.371 | Positive | 552.826 | 878.477 | 22.11 | 20 |
| sp\|Q9UDY4\|DNJB4_HUMAN_IIGYGLPFPK | 29 | 35.371 | Positive | 552.826 | 991.561 | 22.11 | 20 |
| sp\|Q9UJU6\|DBNL_HUMAN_FQDVGPQAPVGSVYQK | 15 | 25 | Positive | 860.438669 | 1173.626249 | 28.7 | 20 |
| sp\|Q9UJU6\|DBNL_HUMAN_FQDVGPQAPVGSVYQK | 15 | 25 | Positive | 860.438669 | 1076.573485 | 28.7 | 20 |
| sp\|Q9UJU6\|DBNL_HUMAN_FQDVGPQAPVGSVYQK | 15 | 25 | Positive | 860.438669 | 948.514908 | 28.7 | 20 |
| sp\|Q9UJU6\|DBNL_HUMAN_FQDVGPQAPVGSVYQK | 15 | 25 | Positive | 860.438669 | 877.477794 | 28.7 | 20 |
| sp\|Q9UJU6\|DBNL_HUMAN_FQDVGPQAPVGSVYQK | 15 | 25 | Positive | 860.438669 | 780.42503 | 28.7 | 20 |
| sp\|Q9UJU6\|DBNL_HUMAN_FQDVGPQAPVGSVYQK | 15 | 25 | Positive | 860.438669 | 681.356616 | 28.7 | 20 |
| sp\|Q9UJU6\|DBNL_HUMAN_FQDVGPQAPVGSVYQK | 15 | 25 | Positive | 860.438669 | 624.335152 | 28.7 | 20 |
| sp\|Q9UJU6\|DBNL_HUMAN_VAGTGEGGLEEMVEELNSGK | 33.985 | 38.985 | Positive | 1003.473 | 518.293 | 37.432 | 20 |
| sp\|Q9UJU6\|DBNL_HUMAN_VAGTGEGGLEEMVEELNSGK | 33.985 | 38.985 | Positive | 1003.473 | 647.335 | 37.432 | 20 |
| sp\|Q9UJU6\|DBNL_HUMAN_VAGTGEGGLEEMVEELNSGK | 33.985 | 38.985 | Positive | 1003.473 | 776.378 | 37.432 | 20 |
| sp\|Q9UJU6\|DBNL_HUMAN_VAGTGEGGLEEMVEELNSGK | 33.985 | 38.985 | Positive | 1003.473 | 875.446 | 37.432 | 20 |
| sp\|Q9UJU6\|DBNL_HUMAN_VAGTGEGGLEEMVEELNSGK | 33.985 | 38.985 | Positive | 1003.473 | 1006.487 | 37.432 | 20 |
| sp\|Q9UJU6\|DBNL_HUMAN_VAGTGEGGLEEMVEELNSGK | 33.985 | 38.985 | Positive | 1003.473 | 1135.529 | 37.432 | 20 |
| sp\|Q9UJU6\|DBNL_HUMAN_VAGTGEGGLEEMVEELNSGK | 33.985 | 38.985 | Positive | 1003.473 | 1264.572 | 37.432 | 20 |

**Table S4**. Modified peptides monitored in urine sample 1. Total areas are calculated as the sum of all heavy-labeled and light peptide areas. Total ratios are calculated by dividing the total heavy by total light area for each peptide.

|  | Replicate 1 | Replicate 1 | Replicate 1 | Replicate 2 | Replicate 2 | Replicate 2 |
| --- | --- | --- | --- | --- | --- | --- |
| **Peptide:** | **H/L Ratio:** | **Heavy area:** | **Light area:** | **H/L Ratio:** | **Heavy area:** | **Light area:** |
|  |  |  |  |  |  |  |
| GGVNDNFQGVLQNVR | 5.34 | 327097 | 61210 | 5.25 | 333916 | 63532 |
| GGVNDNFQGVLQN[+1]VR | 4.41 | 41895 | 9512 | 5.21 | 46247 | 8872 |
| GGVNDNFQGVLQ[+1]NVR | 4.33 | 41025 | 9482 | 5.44 | 50071 | 9197 |
| GGVNDN[+1]FQGVLQNVR | 5.07 | 154534 | 25126 | 4.92 | 156881 | 31900 |
| GGVN[+1]DNFQGVLQNVR | 4.73 | 149265 | 31548 | 5.13 | 149850 | 29214 |
| GGVNDNFQ[+1]GVLQNVR | 5.17 | 139493 | 26975 | 4.37 | 167300 | 38285 |
| GGVNDNFQ[+1]GVLQ[+1]NVR | 3.51 | 25699 | 4080 | 4.22 | 17339 | 1956 |
| GGVN[+1]DN[+1]FQGVLQN[+1]VR | 5.83 | 11914 | 1069 | x | x | x |
| GGVN[+1]DN[+1]FQ[+1]GVLQ[+1]NVR | x | x | x | x | x | x |
| GGVN[+1]DN[+1]FQ[+1]GVLQ[+1]N[+1]VR | x | x | x | x | x | x |
| **Total:** | 5.27 | 890922 | 169002 | 5.04 | 921604 | 182956 |
|  |  |  |  |  |  |  |
|  |  |  |  |  |  |  |
| GFFADYEIPNLQK | 2.87 | 2523994 | 880365 | 2.96 | 2451823 | 829690 |
| GFFADYEIPN[+1]LQK | 2.24 | 552455 | 246994 | 2.21 | 531261 | 240658 |
| GFFADYEIPNLQ[+1]K | 2.16 | 679880 | 314608 | 2.24 | 698400 | 312404 |
| GFFADYEIPN[+1]LQ[+1]K | 1.42 | 137704 | 97285 | 1.52 | 139449 | 92002 |
| **Total:** | 2.53 | 3894033 | 1539252 | 2.59 | 3820933 | 1474754 |
|  |  |  |  |  |  |  |
|  |  |  |  |  |  |  |
| TIVTTLQDSIR | 17.76 | 8198245 | 461707 | 18.42 | 7271878 | 394975 |
| TIVTTLQ[+1]DSIR | 14.12 | 3161409 | 223806 | 14.12 | 2784066 | 197001 |
| **Total:** | 16.57 | 11359654 | 685513 | 16.99 | 10055944 | 591976 |
|  |  |  |  |  |  |  |
|  |  |  |  |  |  |  |
| IQAYDYLEC[+57]SAK | 30.12 | 1439218 | 47822 | 28.01 | 1340032 | 47832 |
| IQ[+1]AYDYLEC[+57]SAK | 12.90 | 272624 | 21122 | 15.15 | 241895 | 15953 |
| Total: | 24.83 | 1711842 | 68944 | 24.80 | 1581927 | 63785 |

**Table S5**. Optimized scheduled SRM methods for heavy-labeled and light peptides.

| Compound | Start Time (min) | End Time (min) | Polarity | Precursor (m/z) | Product (m/z) | Collision Energy (V) | Dwell Time (ms) |
| --- | --- | --- | --- | --- | --- | --- | --- |
| sp\|P02769\|ALBU_BOVIN  HLVDEPQNLIK | 16 | 22.524 | Positive | 653.361701 | 712.435201 | 22.5 | 10 |
| sp\|P02769\|ALBU_BOVIN  HLVDEPQNLIK | 16 | 22.524 | Positive | 653.361701 | 956.504737 | 22.5 | 10 |
| sp\|P02769\|ALBU_BOVIN  HLVDEPQNLIK | 16 | 22.524 | Positive | 653.361701 | 1055.573151 | 22.5 | 10 |
| sp\|P02769\|ALBU_BOVIN  HLVDEPQNLIK | 16 | 22.524 | Positive | 657.368801 | 720.4494 | 22.5 | 10 |
| sp\|P02769\|ALBU_BOVIN  HLVDEPQNLIK | 16 | 22.524 | Positive | 657.368801 | 964.518936 | 22.5 | 10 |
| sp\|P02769\|ALBU_BOVIN  HLVDEPQNLIK | 16 | 22.524 | Positive | 657.368801 | 1063.58735 | 22.5 | 10 |
| sp\|P62745\|RHOB_HUMAN  IQAYDYLEC[+57.0]SAK | 20 | 30 | Positive | 730.839942 | 985.429523 | 24.8 | 30 |
| sp\|P62745\|RHOB_HUMAN  IQAYDYLEC[+57.0]SAK | 20 | 30 | Positive | 730.839942 | 1148.492852 | 24.8 | 30 |
| sp\|P62745\|RHOB_HUMAN  IQAYDYLEC[+57.0]SAK | 20 | 30 | Positive | 730.839942 | 1219.529966 | 24.8 | 30 |
| sp\|P62745\|RHOB_HUMAN  IQ[+1.0]AYDYLEC[+57.0]SAK | 20 | 30 | Positive | 731.33195 | 707.339252 | 24.8 | 30 |
| sp\|P62745\|RHOB_HUMAN  IQ[+1.0]AYDYLEC[+57.0]SAK | 20 | 30 | Positive | 731.33195 | 985.429523 | 24.8 | 30 |
| sp\|P62745\|RHOB_HUMAN  IQ[+1.0]AYDYLEC[+57.0]SAK | 20 | 30 | Positive | 731.33195 | 1219.529966 | 24.8 | 30 |
| sp\|P62745\|RHOB_HUMAN  IQAYDYLEC[+57.0]SAK | 20 | 30 | Positive | 734.847041 | 993.443722 | 24.8 | 30 |
| sp\|P62745\|RHOB_HUMAN  IQAYDYLEC[+57.0]SAK | 20 | 30 | Positive | 734.847041 | 1156.507051 | 24.8 | 30 |
| sp\|P62745\|RHOB_HUMAN  IQAYDYLEC[+57.0]SAK | 20 | 30 | Positive | 734.847041 | 1227.544165 | 24.8 | 30 |
| sp\|P62745\|RHOB_HUMAN  IQ[+1.0]AYDYLEC[+57.0]SAK | 20 | 30 | Positive | 735.339049 | 715.353451 | 24.8 | 30 |
| sp\|P62745\|RHOB_HUMAN  IQ[+1.0]AYDYLEC[+57.0]SAK | 20 | 30 | Positive | 735.339049 | 993.443722 | 24.8 | 30 |
| sp\|P62745\|RHOB_HUMAN  IQ[+1.0]AYDYLEC[+57.0]SAK | 20 | 30 | Positive | 735.339049 | 1227.544165 | 24.8 | 30 |
| sp\|P07996\|TSP1_HUMAN  GGVNDNFQGVLQNVR | 24.738 | 30.738 | Positive | 808.911 | 629.373 | 27 | 30 |
| sp\|P07996\|TSP1_HUMAN  GGVNDNFQGVLQNVR | 24.738 | 30.738 | Positive | 808.911 | 785.463 | 27 | 30 |
| sp\|P07996\|TSP1_HUMAN  GGVNDNFQGVLQNVR | 24.738 | 30.738 | Positive | 808.911 | 913.521 | 27 | 30 |
| sp\|P07996\|TSP1_HUMAN  GGVNDNFQ[+1.0]GVLQNVR | 24.738 | 30.738 | Positive | 809.403 | 629.373 | 27 | 20 |
| sp\|P07996\|TSP1_HUMAN  GGVNDNFQ[+1.0]GVLQNVR | 24.738 | 30.738 | Positive | 809.403 | 785.463 | 27 | 20 |
| sp\|P07996\|TSP1_HUMAN  GGVNDNFQ[+1.0]GVLQNVR | 24.738 | 30.738 | Positive | 809.403 | 914.505 | 27 | 20 |
| sp\|P07996\|TSP1_HUMAN  GGVNDNFQGVLQNVR | 24.738 | 30.738 | Positive | 813.915 | 639.381 | 27 | 30 |
| sp\|P07996\|TSP1_HUMAN  GGVNDNFQGVLQNVR | 24.738 | 30.738 | Positive | 813.915 | 795.471 | 27 | 30 |
| sp\|P07996\|TSP1_HUMAN  GGVNDNFQGVLQNVR | 24.738 | 30.738 | Positive | 813.915 | 923.53 | 27 | 30 |
| sp\|P07996\|TSP1_HUMAN  GGVNDNFQ[+1.0]GVLQNVR | 24.738 | 30.738 | Positive | 814.407 | 639.381 | 27 | 20 |
| sp\|P07996\|TSP1_HUMAN  GGVNDNFQ[+1.0]GVLQNVR | 24.738 | 30.738 | Positive | 814.407 | 795.471 | 27 | 20 |
| sp\|P07996\|TSP1_HUMAN  GGVNDNFQ[+1.0]GVLQNVR | 24.738 | 30.738 | Positive | 814.407 | 924.514 | 27 | 20 |
| sp\|P15104\|GLNA_HUMAN  LVLC[+57.0]EVFK | 27 | 35 | Positive | 504.283346 | 682.322873 | 18 | 30 |
| sp\|P15104\|GLNA_HUMAN  LVLC[+57.0]EVFK | 27 | 35 | Positive | 504.283346 | 795.406937 | 18 | 30 |
| sp\|P15104\|GLNA_HUMAN  LVLC[+57.0]EVFK | 27 | 35 | Positive | 504.283346 | 894.475351 | 18 | 30 |
| sp\|P15104\|GLNA_HUMAN  LVLC[+57.0]EVFK | 27 | 35 | Positive | 508.290445 | 690.337072 | 18 | 30 |
| sp\|P15104\|GLNA_HUMAN  LVLC[+57.0]EVFK | 27 | 35 | Positive | 508.290445 | 803.421136 | 18 | 30 |
| sp\|P15104\|GLNA_HUMAN  LVLC[+57.0]EVFK | 27 | 35 | Positive | 508.290445 | 902.48955 | 18 | 30 |
| sp\|P02769\|ALBU_BOVIN  LGEYGFQNALIVR | 30.5 | 34.5 | Positive | 740.401358 | 1017.58399 | 25.1 | 10 |
| sp\|P02769\|ALBU_BOVIN  LGEYGFQNALIVR | 30.5 | 34.5 | Positive | 740.401358 | 813.494113 | 25.1 | 10 |
| sp\|P02769\|ALBU_BOVIN  LGEYGFQNALIVR | 30.5 | 34.5 | Positive | 740.401358 | 685.435535 | 25.1 | 10 |
| sp\|P02769\|ALBU_BOVIN  LGEYGFQNALIVR | 30.5 | 34.5 | Positive | 745.405492 | 1027.592259 | 25.1 | 10 |
| sp\|P02769\|ALBU_BOVIN  LGEYGFQNALIVR | 30.5 | 34.5 | Positive | 745.405492 | 823.502382 | 25.1 | 10 |
| sp\|P02769\|ALBU_BOVIN  LGEYGFQNALIVR | 30.5 | 34.5 | Positive | 745.405492 | 695.443804 | 25.1 | 10 |
| sp\|O75608\|LYPA1_HUMAN  LAGVTALSC[+57.0]WLPLR | 39.748 | 50 | Positive | 778.934511 | 385.25578 | 26.3 | 30 |
| sp\|O75608\|LYPA1_HUMAN  LAGVTALSC[+57.0]WLPLR | 39.748 | 50 | Positive | 778.934511 | 931.481834 | 26.3 | 30 |
| sp\|O75608\|LYPA1_HUMAN  LAGVTALSC[+57.0]WLPLR | 39.748 | 50 | Positive | 778.934511 | 1044.565898 | 26.3 | 30 |
| sp\|O75608\|LYPA1_HUMANLAGVTALSC[+57.0]WLPLR | 39.748 | 50 | Positive | 783.938645 | 395.264049 | 26.3 | 30 |
| sp\|O75608\|LYPA1_HUMANLAGVTALSC[+57.0]WLPLR | 39.748 | 50 | Positive | 783.938645 | 941.490103 | 26.3 | 30 |
| sp\|O75608\|LYPA1_HUMANLAGVTALSC[+57.0]WLPLR | 39.748 | 50 | Positive | 783.938645 | 1054.574167 | 26.3 | 30 |
| sp\|P55268\|LAMB2_HUMANGSC[+57.0]YPATGDLLVGR | 18 | 28.184 | Positive | 733.359 | 729.425 | 25 | 20 |
| sp\|P55268\|LAMB2_HUMANGSC[+57.0]YPATGDLLVGR | 18 | 28.184 | Positive | 733.359 | 998.563 | 25 | 20 |
| sp\|P55268\|LAMB2_HUMANGSC[+57.0]YPATGDLLVGR | 18 | 28.184 | Positive | 733.359 | 1161.626 | 25 | 20 |
| sp\|P55268\|LAMB2_HUMANGSC[+57.0]YPATGDLLVGR | 18 | 28.184 | Positive | 738.363 | 739.434 | 25 | 20 |
| sp\|P55268\|LAMB2_HUMANGSC[+57.0]YPATGDLLVGR | 18 | 28.184 | Positive | 738.363 | 1008.571 | 25 | 20 |
| sp\|P55268\|LAMB2_HUMANGSC[+57.0]YPATGDLLVGR | 18 | 28.184 | Positive | 738.363 | 1171.635 | 25 | 20 |
| sp\|P02769\|ALBU_BOVIN  LVNELTEFAK | 23 | 29.18 | Positive | 582.319 | 595.309 | 20 | 10 |
| sp\|P02769\|ALBU_BOVIN  LVNELTEFAK | 23 | 29.18 | Positive | 582.319 | 708.393 | 20 | 10 |
| sp\|P02769\|ALBU_BOVIN  LVNELTEFAK | 23 | 29.18 | Positive | 582.319 | 951.478 | 20 | 10 |
| sp\|P02769\|ALBU_BOVIN  LVNELTEFAK | 23 | 29.18 | Positive | 586.326 | 603.323 | 20 | 10 |
| sp\|P02769\|ALBU_BOVIN  LVNELTEFAK | 23 | 29.18 | Positive | 586.326 | 716.407 | 20 | 10 |
| sp\|P02769\|ALBU_BOVIN  LVNELTEFAK | 23 | 29.18 | Positive | 586.326 | 959.492 | 20 | 10 |
| sp\|P07996\|TSP1_HUMAN  TIVTTLQDSIR | 24.362 | 33 | Positive | 623.853709 | 832.452307 | 23.4 | 30 |
| sp\|P07996\|TSP1_HUMAN  TIVTTLQDSIR | 24.362 | 33 | Positive | 623.853709 | 933.499986 | 23.4 | 30 |
| sp\|P07996\|TSP1_HUMAN  TIVTTLQDSIR | 24.362 | 33 | Positive | 623.853709 | 1032.5684 | 23.4 | 30 |
| sp\|P07996\|TSP1_HUMAN  TIVTTLQ[+1.0]DSIR | 24.362 | 33 | Positive | 624.345717 | 833.436323 | 23.4 | 30 |
| sp\|P07996\|TSP1_HUMAN  TIVTTLQ[+1.0]DSIR | 24.362 | 33 | Positive | 624.345717 | 934.484002 | 23.4 | 30 |
| sp\|P07996\|TSP1_HUMAN  TIVTTLQ[+1.0]DSIR | 24.362 | 33 | Positive | 624.345717 | 1033.552416 | 23.4 | 30 |
| sp\|P07996\|TSP1_HUMAN  TIVTTLQDSIR | 24.362 | 33 | Positive | 628.857844 | 842.460576 | 23.4 | 30 |
| sp\|P07996\|TSP1_HUMAN  TIVTTLQDSIR | 24.362 | 33 | Positive | 628.857844 | 943.508255 | 23.4 | 30 |
| sp\|P07996\|TSP1_HUMAN  TIVTTLQDSIR | 24.362 | 33 | Positive | 628.857844 | 1042.576669 | 23.4 | 30 |
| sp\|P07996\|TSP1_HUMAN  TIVTTLQ[+1.0]DSIR | 24.362 | 33 | Positive | 629.349852 | 843.444592 | 23.4 | 30 |
| sp\|P07996\|TSP1_HUMAN  TIVTTLQ[+1.0]DSIR | 24.362 | 33 | Positive | 629.349852 | 944.492271 | 23.4 | 30 |
| sp\|P07996\|TSP1_HUMAN  TIVTTLQ[+1.0]DSIR | 24.362 | 33 | Positive | 629.349852 | 1043.560685 | 23.4 | 30 |
| sp\|Q10588\|BST1_HUMAN  GFFADYEIPNLQK | 31 | 37.016 | Positive | 771.385373 | 841.477794 | 26 | 20 |
| sp\|Q10588\|BST1_HUMAN  GFFADYEIPNLQK | 31 | 37.016 | Positive | 771.385373 | 712.435201 | 26 | 20 |
| sp\|Q10588\|BST1_HUMAN  GFFADYEIPNLQK | 31 | 37.016 | Positive | 771.385373 | 599.351137 | 26 | 20 |
| sp\|Q10588\|BST1_HUMAN  GFFADYEIPNLQK | 31 | 37.016 | Positive | 775.392473 | 849.491993 | 26 | 20 |
| sp\|Q10588\|BST1_HUMAN  GFFADYEIPNLQK | 31 | 37.016 | Positive | 775.392473 | 720.4494 | 26 | 20 |
| sp\|Q10588\|BST1_HUMAN  GFFADYEIPNLQK | 31 | 37.016 | Positive | 775.392473 | 607.365336 | 26 | 20 |
| sp\|Q10588\|BST1_HUMAN  GFFADYEIPN[+1.0]LQ[+1.0]K | 31 | 37.016 | Positive | 772.369389 | 1121.536097 | 28.8 | 20 |
| sp\|Q10588\|BST1_HUMAN  GFFADYEIPN[+1.0]LQ[+1.0]K | 31 | 37.016 | Positive | 772.369389 | 714.403233 | 28.8 | 20 |
| sp\|Q10588\|BST1_HUMAN  GFFADYEIPN[+1.0]LQ[+1.0]K | 31 | 37.016 | Positive | 772.369389 | 601.319169 | 28.8 | 20 |
| sp\|Q10588\|BST1_HUMAN  GFFADYEIPN[+1.0]LQ[+1.0]K | 31 | 37.016 | Positive | 776.376489 | 1129.550296 | 28.8 | 20 |
| sp\|Q10588\|BST1_HUMAN  GFFADYEIPN[+1.0]LQ[+1.0]K | 31 | 37.016 | Positive | 776.376489 | 722.417432 | 28.8 | 20 |
| sp\|Q10588\|BST1_HUMAN  GFFADYEIPN[+1.0]LQ[+1.0]K | 31 | 37.016 | Positive | 776.376489 | 609.333368 | 28.8 | 20 |
| sp\|Q10588\|BST1_HUMAN  GFFADYEIPN[+1.0]LQK | 31 | 37.016 | Positive | 771.877381 | 713.419217 | 26.1 | 20 |
| sp\|Q10588\|BST1_HUMAN  GFFADYEIPN[+1.0]LQK | 31 | 37.016 | Positive | 771.877381 | 600.335153 | 26.1 | 20 |
| sp\|Q10588\|BST1_HUMAN  GFFADYEIPN[+1.0]LQK | 31 | 37.016 | Positive | 771.877381 | 503.282389 | 26.1 | 20 |
| sp\|Q10588\|BST1_HUMAN  GFFADYEIPN[+1.0]LQK | 31 | 37.016 | Positive | 775.884481 | 721.433416 | 26.1 | 20 |
| sp\|Q10588\|BST1_HUMAN  GFFADYEIPN[+1.0]LQK | 31 | 37.016 | Positive | 775.884481 | 608.349352 | 26.1 | 20 |
| sp\|Q10588\|BST1_HUMAN  GFFADYEIPN[+1.0]LQK | 31 | 37.016 | Positive | 775.884481 | 511.296588 | 26.1 | 20 |
| sp\|Q10588\|BST1_HUMAN  GFFADYEIPNLQ[+1.0]K | 31 | 37.016 | Positive | 771.877381 | 1120.552081 | 26.1 | 20 |
| sp\|Q10588\|BST1_HUMAN  GFFADYEIPNLQ[+1.0]K | 31 | 37.016 | Positive | 771.877381 | 713.419217 | 26.1 | 20 |
| sp\|Q10588\|BST1_HUMAN  GFFADYEIPNLQ[+1.0]K | 31 | 37.016 | Positive | 771.877381 | 600.335153 | 26.1 | 20 |
| sp\|Q10588\|BST1_HUMAN  GFFADYEIPNLQ[+1.0]K | 31 | 37.016 | Positive | 775.884481 | 1128.56628 | 26.1 | 20 |
| sp\|Q10588\|BST1_HUMAN  GFFADYEIPNLQ[+1.0]K | 31 | 37.016 | Positive | 775.884481 | 721.433416 | 26.1 | 20 |
| sp\|Q10588\|BST1_HUMAN  GFFADYEIPNLQ[+1.0]K | 31 | 37.016 | Positive | 775.884481 | 608.349352 | 26.1 | 20 |

**Table S6.** Associations between urine excretion rate of Ang II-regulated proteins and clinical characteristics such as sex, age and disease group. Numbers represent p-values. For variable sex, two sample t-test was performed. For age, t-test for correlation testing was performed. For disease groups, ANOVA was used. *p<0.05 **p<0.01***p<0.001.

| **Protein** | **Sex** | **Age** | **Disease group** |
| --- | --- | --- | --- |
| BST1_HUMAN | 0.048138131* | 0.428927966 | 0.008081373** |
| GLNA_HUMAN | 1.10E-05*** | 0.318632437 | 0.073735393 |
| LAMB2_HUMAN | 0.128170096 | 0.502380473 | 0.020743474* |
| LYPA1_HUMAN | 0.004753231** | 0.215451837 | 0.011589833* |
| RHOB_HUMAN | 0.012193125* | 0.395149383 | 0.020608354* |
| TSP1_HUMAN_GGV | 0.019179623* | 0.454168448 | 0.012624328* |
| TSP1_HUMAN_TIV | 0.005861328** | 0.535154461 | 0.019311872* |

**Figure S1.** Calibration curves for 7 Ang II-regulated peptides. A) TSP1 peptide TIVTTLQDSIR. y = -0.2239 + 0.2930 x; R^2^ = 0.9997; B) TSP1 peptide GGVNDFQGVLQNVR. y = 0.06662 + 0.1242 x; R^2^ = 0.999; C) GLUL peptide. y = 0.08380 + 0.09353 x; R^2^= 0.9969; D) RHOB peptide. y = 0.3098 + 0.2902 x; R^2^ = 0.9935; E) BST1 peptide. y = 0.05329 + 0.03982x; R^2^= 0.9995; F) LYPLA1 peptide. y = 0.2281 + 0.1405 x; R^2^ = 0.9794; G) LAMB2 peptide. y = -0.2221 + 0.2719 x; R^2^ = 0.9998. Values at the low range of the curve are shown on the top left.

A)


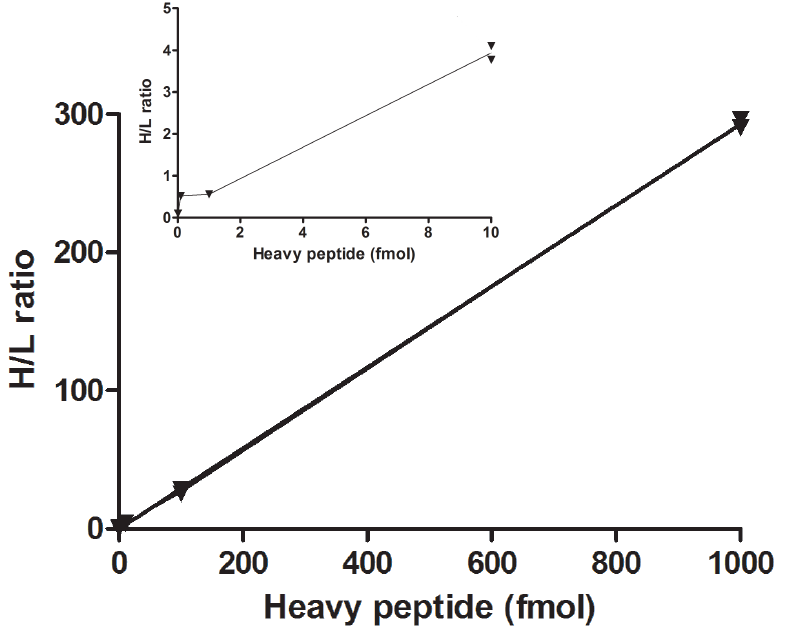


B)


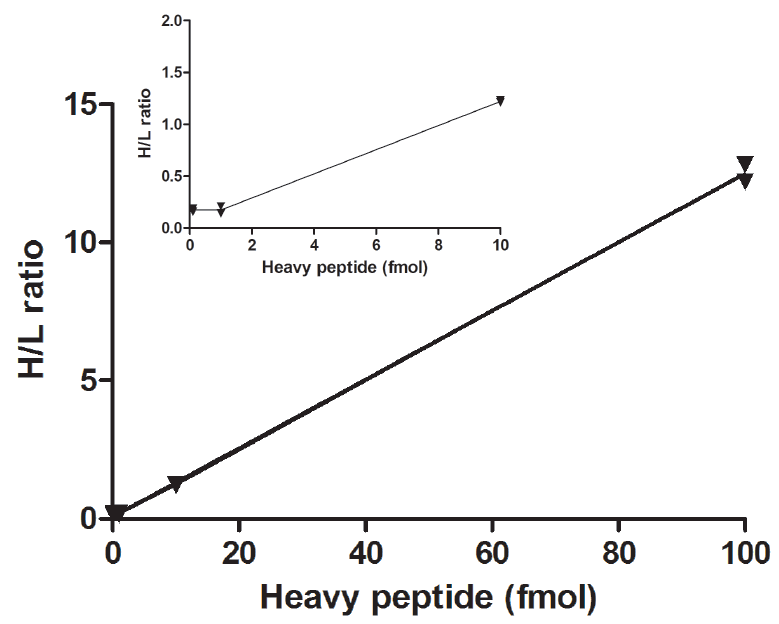


C)


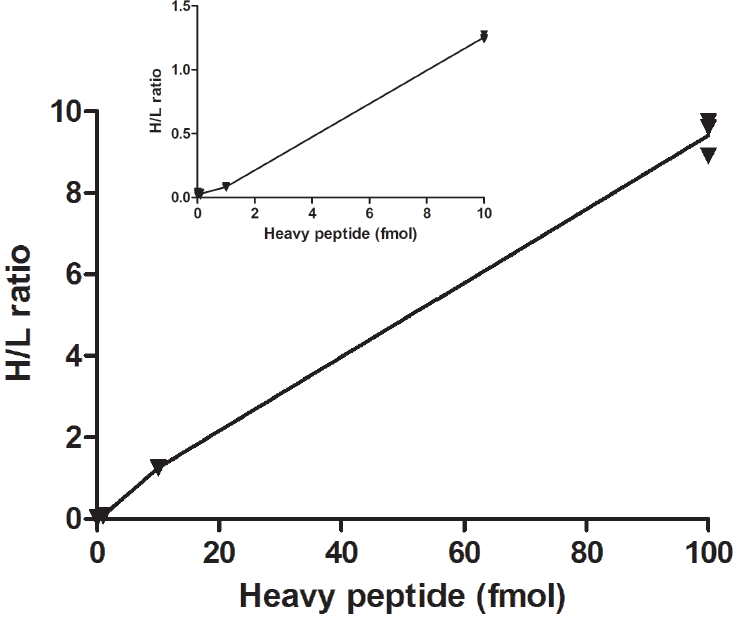


D)


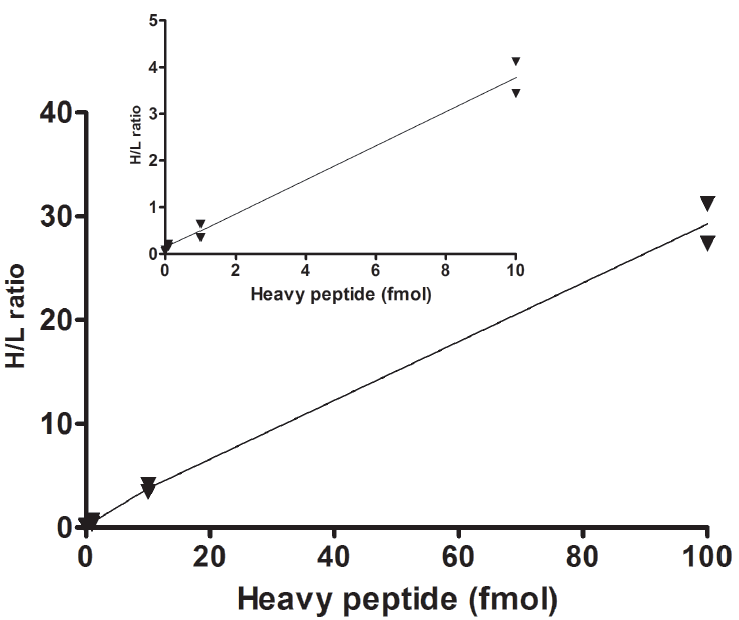


E)


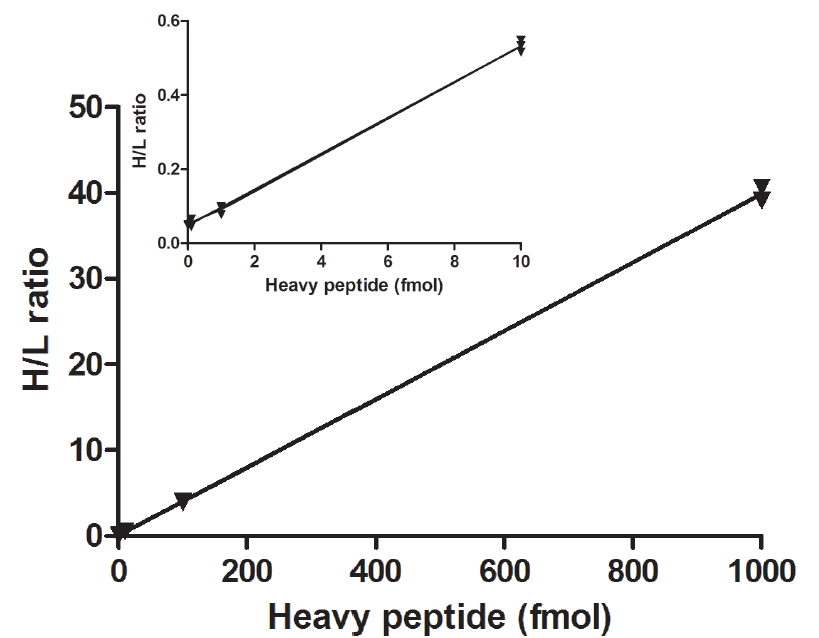


F)


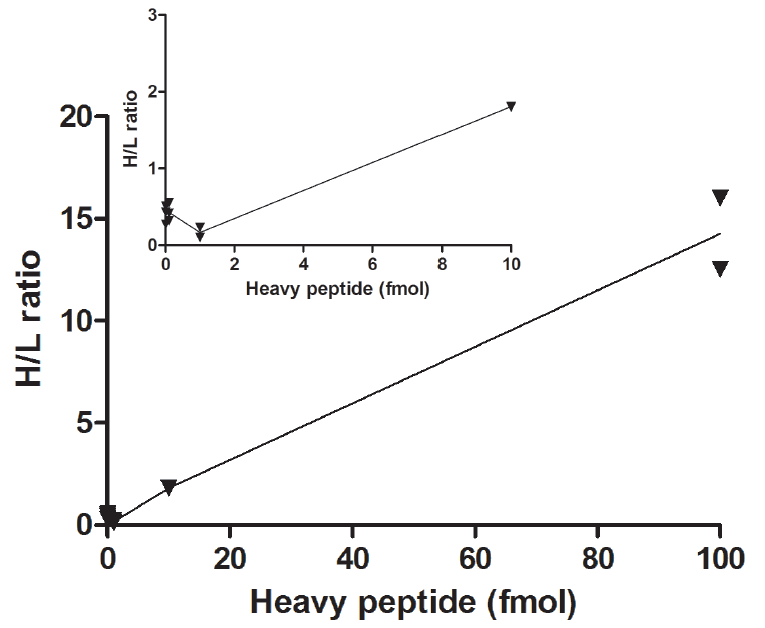


G)


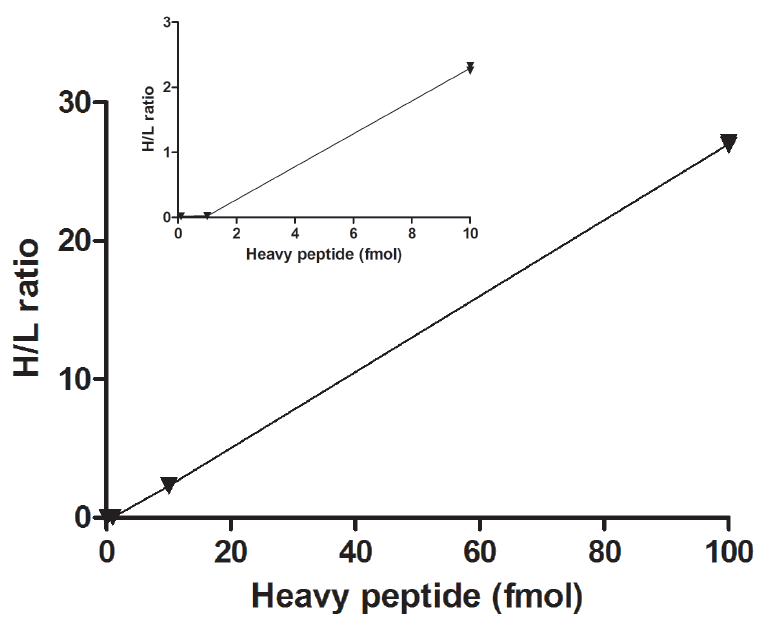


**Figure S2**. Between-run variability of L/H peptide ratios adjusted for L/H BSA ratios in different **A)** urine samples (each box represents one urine sample) and **B)** peptides (each box represents one peptide). X-axis represents log2-transformed adjusted L/H ratios in urine sample A, divided by the L/H ratio in urine sample B. Samples A and B represent aliquots of the same urine sample that were thawed and processed on different days. Log2-transformed ratios thus represent a measure of between-run variability, and perfectly reproducible measurements between samples A and B would have a log2-transformed ratio of 0.

**A)**

**
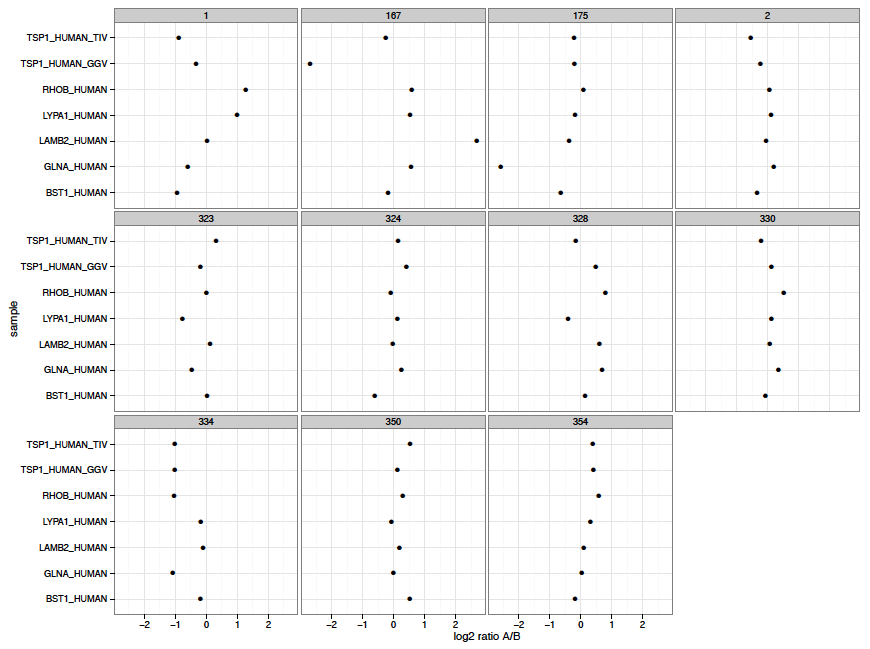
**

**B)**

**
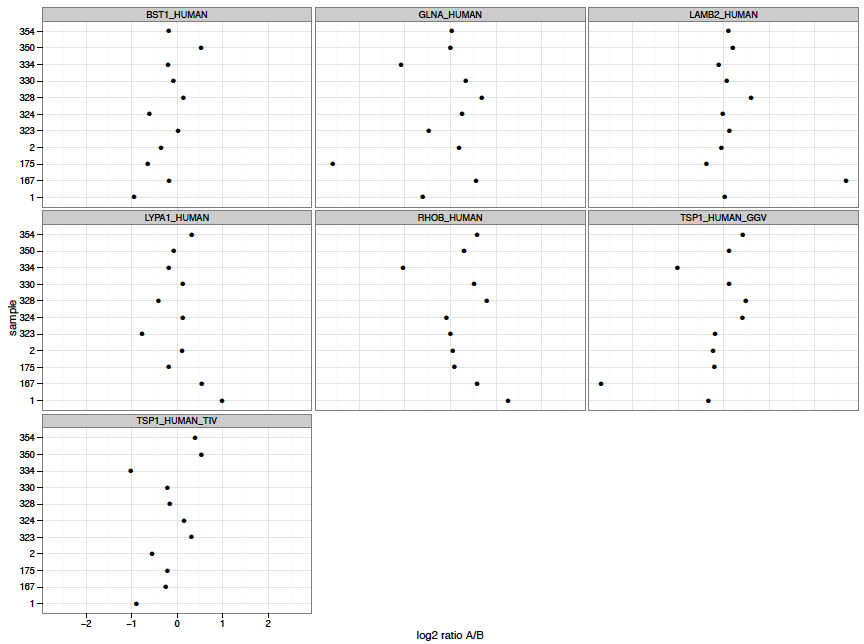
**

**Figure S3.** Linear correlation between urine excretion rates of two peptides of TSP1. Peptide 1 measurements are presented on x-axis, while peptide 2 measurements are on y-axis.

**Figure S4.** Protein-protein interaction network including the 6 monitored proteins and their direct interaction partners and interaction among them. Physical protein interactions obtained from IID v2016-03 (26516188), and visualization performed in NAViGaTOR 2.3 (19837718). Query proteins are highlighted as rectangles. Nodes with red signify the shortest path connection among query proteins. Node color represent biological function, as per legend. Corresponding XML file can be accessed at: [http://www.cs.utoronto.ca/~juris/data/ClinProteom16/](http://www.cs.utoronto.ca/%7Ejuris/data/ClinProteom16/)

**
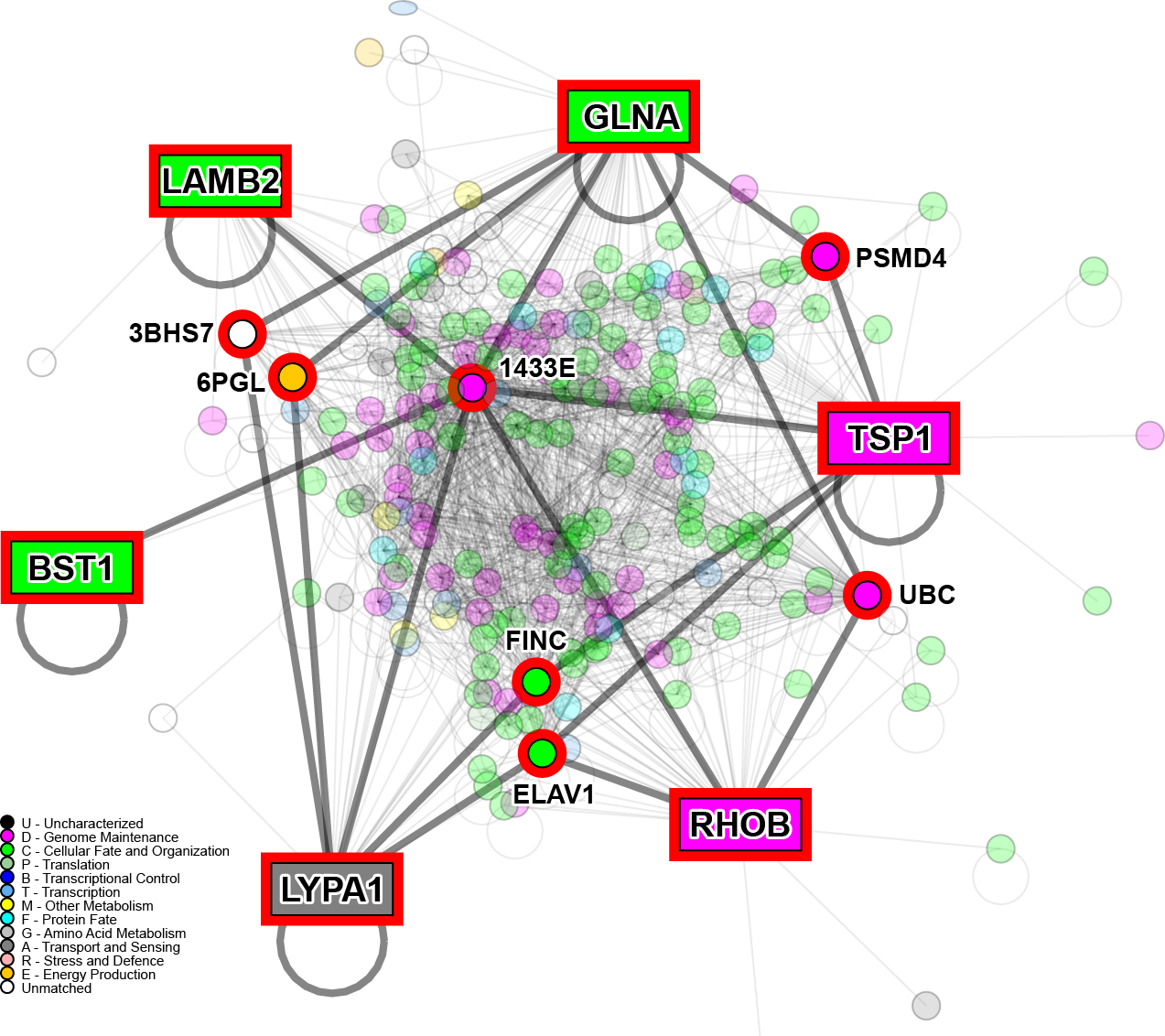
**

**Figure S5.** Pathway enrichment analysis among the 6 monitored proteins and their PPI partners from Figure S4 using pathDIP ver. 1.0. Only top 60 most significantly enriched pathways taken from sources listed to the right of the graph are displayed. Note, p-values representing significance of some of these pathways were equal to zero, to allow their log10 transformation for visualization purposes, those were adjusted to 10^-18^.


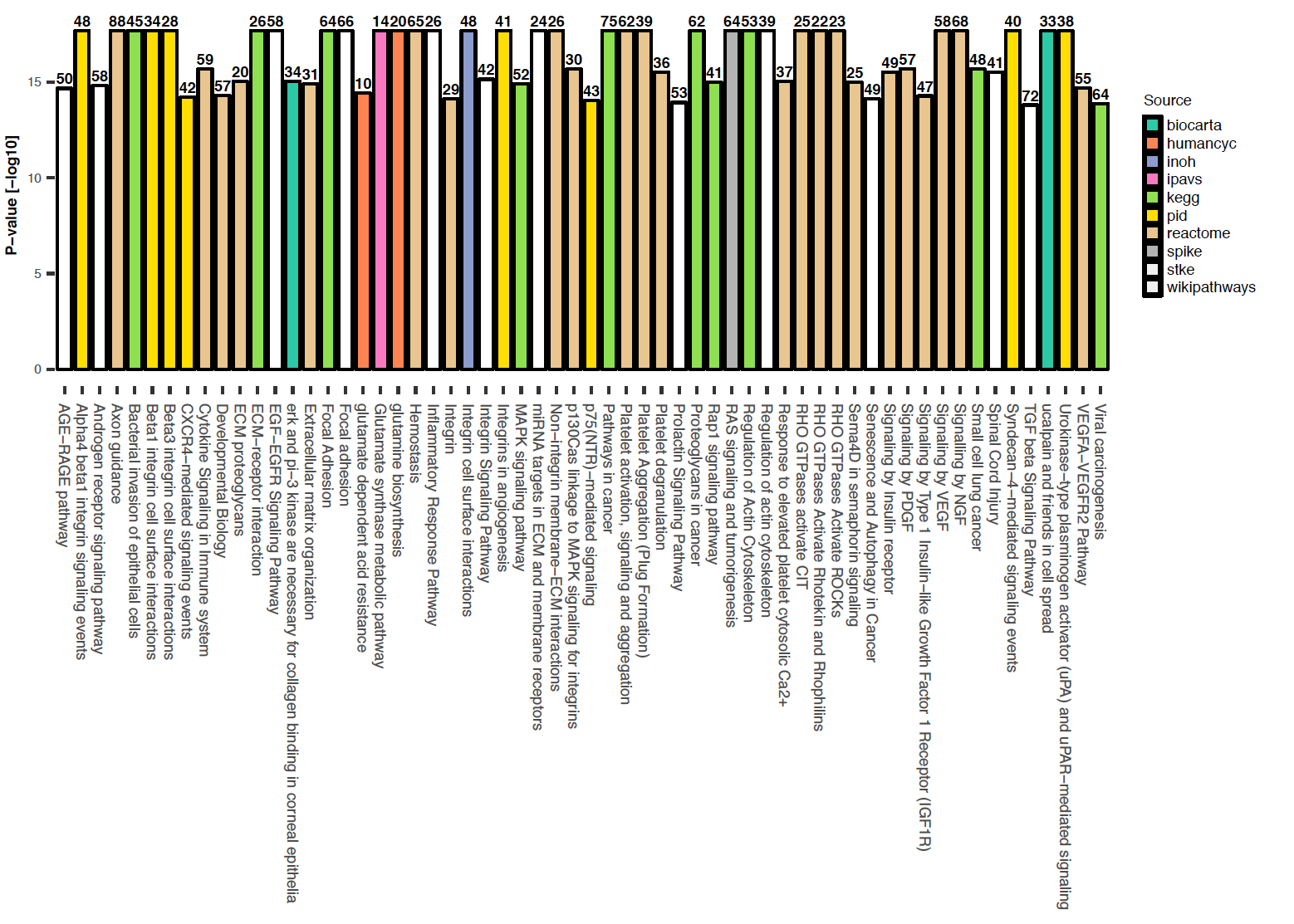

Supplement: Supplementary file 1 — 10.1186/s12014-016-9117-x Table S1. Characteristics of PCKD patients and healthy controls. *Estimated glomerular filtration rate (eGFR) was calculated from MDRD equation or 24-hour urine creatinine excretion rate. ψTotal kidney volume was calculated by magnetic resonance imaging (MRI). Table S2. Parameters of a multiplex scheduled SRM assay of all monitored peptides, including peptide sequence, m/z, modifications monitored and charge. Table S3. Scheduled SRM parameters of light peptides monitored during method development. Table S4. Modified peptides monitored in urine sample 1. Total areas are calculated as the sum of all heavy-labeled and light peptide areas. Total ratios are calculated by dividing the total heavy by total light area for each peptide. Table S5. Optimized scheduled SRM methods for heavy-labeled and light peptides. Table S6. Associations between urine excretion rate of Ang II-regulated proteins and clinical characteristics such as sex, age and disease group. Numbers represent p-values. For variable sex, two sample t-test was performed. For age, t-test for correlation testing was performed. For disease groups, ANOVA was used. *p<0.05 **p<0.01***p<0.001. Additional file 1: Figure S1. Calibration curves for 7 Ang II-regulated peptides. A) TSP1 peptide TIVTTLQDSIR. y = -0.2239 + 0.2930 x; R2 = 0.9997; B) TSP1 peptide GGVNDFQGVLQNVR. y = 0.06662 + 0.1242 x; R2 = 0.999; C) GLUL peptide. y = 0.08380 + 0.09353 x; R2= 0.9969; D) RHOB peptide. y = 0.3098 + 0.2902 x; R2 = 0.9935; E) BST1 peptide. y = 0.05329 + 0.03982x; R2= 0.9995; F) LYPLA1 peptide. y = 0.2281 + 0.1405 x; R2 = 0.9794; G) LAMB2 peptide. y = -0.2221 + 0.2719 x; R2 = 0.9998. Values at the low range of the curve are shown on the top left. Figure S2. Between-run variability of L/H peptide ratios adjusted for L/H BSA ratios in different A) urine samples (each box represents one urine sample) and B) peptides (each box represents one peptide). X-axis represents log2-transformed adjusted L/H ratios in [file 12014_2016_9117_MOESM1_ESM.docx]
